# Transport of Electrons in Tangled Magnetic Fields

**DOI:** 10.1007/s11214-026-01303-9

**Published:** 2026-05-23

**Authors:** Daniel Verscharen, Natasha Jeffrey, Anton Artemyev, Jesse T. Coburn, Matthew W. Kunz, Oreste Pezzi, Mario Riquelme, Ida Svenningsson, Lynn B. Wilson III

**Affiliations:** 1 Mullard Space Science Laboratory, University College London, Holmbury House, Dorking, RH5 6NT UK; 2 Department of Mathematics, Physics and Electrical Engineering, Northumbria University, Newcastle upon Tyne, NE1 8ST UK; 3 Department of Earth, Planetary, and Space Sciences, University of California, Los Angeles, CA 90095 USA; 4 Department of Astrophysical Sciences, Princeton University, Princeton, NJ 08544 USA; 5 Princeton Plasma Physics Laboratory, Princeton, NJ 08543 USA; 6 Institute for Plasma Science and Technology, National Research Council of Italy, G. Amendola 122/D, Bari, I-70126 Italy; 7 Departamento de Física, Facultad de Ciencias Físicas y Matemáticas (FCFM), Universidad de Chile, Beauchef 850, Santiago, Chile; 8 Swedish Institute of Space Physics, Uppsala, 751 21 Sweden; 9 Department of Physics and Astronomy, Uppsala University, Uppsala, 751 20 Sweden; 10 Department of Physics, Chalmers University of Technology, Gothenburg, 412 96 Sweden; 11 Heliophysics Division, NASA Goddard Space Flight Center, Code 672, Bldg. 21, Rm. 143A, Greenbelt, MD 20771 USA; 12 Present Address: Laboratoire de Physique des Plasmas, CNRS, Observatoire de Paris, Sorbonne Université, Université Paris Saclay, École polytechnique, Institut Polytechnique de Paris, Palaiseau, 91128 France

**Keywords:** Heliophysics, Electron-astrophysics, Particle trapping, Wave–particle interactions, Diffusion, Magnetic fields

## Abstract

Cosmic magnetic fields are typically inhomogeneous and often highly tangled due to large-scale plasma flows, turbulence, and instabilities. If the variations in the magnetic field occur on scales that are large compared to the gyro-radius of the plasma electrons, the electrons are primarily confined to gyro-centre trajectories along the field lines. Therefore, in-situ electron measurements help us map out the connectivity of the magnetic field in space plasmas. Gyro-centre drifts, wave–particle interactions, trapping, and cross-field diffusion are processes related to field inhomogeneities and fluctuations; they have the potential to modify or even disrupt the transport of electrons along field lines. We introduce the basic principles of electron transport in tangled magnetic fields and review the creation of tangled fields through turbulence and instabilities as well as the modulation of parallel electron transport through kinetic instabilities. We then describe trapping and de-trapping effects in inhomogeneous magnetic fields, as well as electron diffusion and energisation across the magnetic field. The transport of electrons in tangled fields results from a complex interplay of plasma processes that occur on a broad range of scales. A combination of in-situ plasma measurements, remote-sensing plasma observations, and plasma theory and simulations is required to resolve this contemporary challenge to the fields of heliophysics and astrophysics.

## Introduction

In a homogeneous, static magnetic field $\boldsymbol{B}$, the Lorentz force deflects electrons in the direction perpendicular to their velocity vector $\boldsymbol{v}$ and to $\boldsymbol{B}$. Consequently, the electrons undergo gyration at their cyclotron frequency

1 $$ \Omega _{\mathrm {e}}=\frac{eB}{m_{\mathrm {e}}c}, $$

where $e<0$ is the charge of an electron, $m_{\mathrm {e}}$ is the mass of an electron, and $c$ is the speed of light.^1^ In the absence of an electric field, the particles conserve their kinetic energy and spiral about the magnetic field $\boldsymbol{B}$. In a reference frame in which the centre of this spiral motion is at rest, we denote the magnitude of the velocity vector in the plane perpendicular to $\boldsymbol{B}$ as $v_{\perp}$ and the component of the velocity vector parallel to $\boldsymbol{B}$ as $v_{\parallel}$. If the field is homogeneous and no other forces act on the electron, $v_{\perp}$ and $v_{\parallel}$ are constant. The radius of the spiral trajectory is given by the gyro-radius

2 $$ \rho _{\mathrm {e}}=\frac{v_{\perp}}{|\Omega _{\mathrm {e}}|}. $$

In most space and astrophysical plasmas, $\rho _{\mathrm {e}}$ is significantly smaller than the characteristic scales of the system (Verscharen et al. 2019), so that gyration confines individual electrons into cylindrical volumes with small radii that are each centred on a given magnetic-field line, and the electrons are *magnetised*. While the radii of the accessible cylindrical volumes are small, the electrons are free to move parallel to $\boldsymbol{B}$. For this reason, most electron transport occurs along the magnetic field.

All space and astrophysical plasma systems ultimately break the assumptions of a homogeneous and static magnetic field though. Large-scale flows, instabilities, and turbulence tangle magnetic-field lines, leading to complex field geometries (Bruno and Carbone 2013; Laitinen et al. 2023). Even though electron transport still occurs mostly along the magnetic field, additional effects modify the electron motion compared to the homogeneous case. Since most tangling processes occur on timescales that are large compared to the associated electron gyro-motion (i.e., $\gg 1/|\Omega _{\mathrm {e}}|$), we focus on spatial inhomogeneity in this article while assuming that the field is quasi-static in time.

**Fig. 1:**
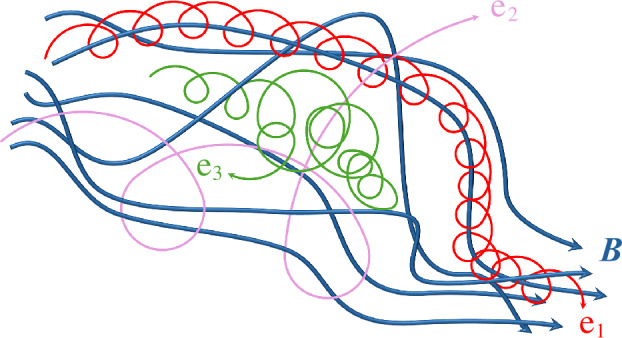
Example trajectories of electrons in tangled magnetic-field lines ($\boldsymbol{B}$, blue). Electron 1 (e_1_, red) follows a given field line. Electron 2 (e_2_, purple) diffuses across field lines. Electron 3 (e_3_, green) undergoes reflection at a mirror point

Figure 1 illustrates three example electron trajectories in tangled magnetic fields. The field lines are indicated in blue. Electron 1 (e_1_, red) has a small $\rho _{\mathrm {e}}$ compared to the scales over which $\boldsymbol{B}$ changes. Therefore, e_1_ gyrates about a given field line and follows this field line when averaged over the electron’s gyro-motion. The path length of the gyro-centre trajectory of a magnetised electron between two given points is longer if the field line is tangled rather than straight between both points.

The magnetic moment

3 $$ \mu =\frac{m_{\mathrm {e}}v_{\perp}^{2}}{2B} $$

of e_1_ remains constant along the trajectory of the particle. As long as the electric field is negligible along the electron trajectory, the electron kinetic energy

4 $$ W=\frac{1}{2}m_{\mathrm {e}}\left (v_{\perp}^{2}+v_{\parallel}^{2} \right ) $$

also remains constant. In the presence of an electric field $\boldsymbol{E}$ with a component perpendicular to the magnetic field or a large-scale (compared to $\rho _{\mathrm {e}}$) inhomogeneity of the magnetic field, the gyro-centre of the gyrating electron follows the guiding-centre motion with the velocity (Hazeltine 1973; Chen 2019)

5 $$ \boldsymbol{V}_{\mathrm {g}}=c \frac{\boldsymbol{E}\,\text{{$\boldsymbol{{\times }}$}}\,\boldsymbol{B}}{B^{2}}+ \frac{\mu}{m_{\mathrm {e}}\Omega _{\mathrm {e}}}\hat{\boldsymbol{b}}\, \text{{$\boldsymbol{{\times }}$}}\,\text{{$\boldsymbol{{\nabla }}$}}B+ \frac{v_{\parallel}^{2}}{\Omega _{\mathrm {e}}}\hat{\boldsymbol{b}}\, \text{{$\boldsymbol{{\times }}$}}\,\left (\hat{\boldsymbol{b}}\, \text{{$\boldsymbol{{\cdot }}$}}\,\text{{$\boldsymbol{{\nabla }}$}}\right ) \hat{\boldsymbol{b}}+v_{\parallel}\hat{\boldsymbol{b}}, $$

where $\hat{\boldsymbol{b}}=\boldsymbol{B}/B$. The magnetic moment $\mu $ is here measured in a reference frame that drifts with the $\boldsymbol{E}\,\text{{$\boldsymbol{{\times }}$}}\,\boldsymbol{B}$ drift, leading to the definition

6 $$ v_{\perp}^{2}=\left (\boldsymbol{v}-v_{\parallel}\hat{\boldsymbol{b}}-c \frac{\boldsymbol{E}\,\text{{$\boldsymbol{{\times }}$}}\,\boldsymbol{B}}{B^{2}} \right )^{2}, $$

where $v_{\parallel}=\boldsymbol{v}\,\text{{$\boldsymbol{{\cdot }}$}}\, \hat{\boldsymbol{b}}$. In Eq. (5), the first term describes the $\boldsymbol{E}\,\text{{$\boldsymbol{{\times }}$}}\,\boldsymbol{B}$ drift. In a system in which the frozen-in condition holds, the field lines are advected at the speed $c\boldsymbol{E}\,\text{{$\boldsymbol{{\times }}$}}\,\boldsymbol{B}/B^{2}$ (see also Sect. 2.1.1), so that the $\boldsymbol{E}\,\text{{$\boldsymbol{{\times }}$}}\,\boldsymbol{B}$ drift typically conserves the association of a gyrating electron with its magnetic-field line. The second term in Eq. (5) describes the gradient drift. It results from a spatial variation in the gyro-radius of the electron. When during its gyro-motion the electron is exposed to spatial variation in the magnitude of $B$, its gyro-radius is smaller (at times of larger $B$) or larger (at times of smaller $B$). This variation causes the electron to drift in the direction of $-\hat{\boldsymbol{b}}\,\text{{$\boldsymbol{{\times }}$}}\, \text{{$\boldsymbol{{\nabla }}$}}B$, given that $\Omega _{\mathrm {e}}<0$. The third term in Eq. (5) describes the curvature drift. It results from the motion of the electron along a curved magnetic-field line. An electron following a curved field line experiences a centrifugal force directed perpendicular to the curved field line, which alternately accelerates and decelerates the gyro-motion. Therefore, an electron drifts in the direction $-\hat{\boldsymbol{b}}\,\text{{$\boldsymbol{{\times }}$}}\,( \hat{\boldsymbol{b}}\,\text{{$\boldsymbol{{\cdot }}$}}\, \text{{$\boldsymbol{{\nabla }}$}})\hat{\boldsymbol{b}}$, i.e., perpendicular to $\boldsymbol{B}$ and the curvature vector $\boldsymbol{\kappa }=(\hat{\boldsymbol{b}}\, \text{{$\boldsymbol{{\cdot }}$}}\,\text{{$\boldsymbol{{\nabla }}$}}) \hat{\boldsymbol{b}}$. The fourth term in Eq. (5) describes the motion of the electron along the magnetic field. The gradient and curvature drifts always occur in combination and allow electrons to leave their associated magnetic-field lines. They play a key role, for example, in laboratory plasmas in which particle drifts off the associated field lines can lead to loss of plasma confinement, unless the field geometry returns particles to the plasma core.

Electron 2 (e_2_, purple) has a greater $v_{\perp}$ compared to e_1_. Therefore, its gyro-radius $\rho _{\mathrm {e}}$ is larger than the gyro-radius of e_1_ at the same $B$. The electron still undergoes gyration in regions of large $B$ as seen at the beginning of its shown trajectory. Later, it encounters regions with variation in $\boldsymbol{B}$ on scales that are comparable to or smaller than its $\rho _{\mathrm {e}}$. There, the electron loses its strong magnetisation and moves, while still undergoing some deflections due to the Lorentz force, by some distance across the field. Its magnetic moment $\mu $ is not conserved because the electron encounters a significant change in $\boldsymbol{B}$ during individual gyro-orbits. The particle undergoes spatial diffusion across the magnetic field. In non-static field configurations, a fast time variation of the magnetic field can also contribute to the breaking of $\mu $-conservation.

Electron 3 (e_3_, green) represents a magnetised particle that travels from a region of low $B$ into a region of high $B$. Due to the conservation of $\mu $ and $W$, the electron’s $v_{\perp}$ increases and its $v_{\parallel}$ decreases as $B$ increases. The gyro-centre of the electron propagates to a point where $v_{\parallel}=0$. This point is called the *mirror point*. The gyrating electron undergoes reflection at this point, and its gyro-centre then propagates back into the region of lower $B$. If the gyrating electron encounters another region with higher $B$ after propagating through the location of lowest $B$ along its trajectory, it may undergo consecutive reflections between both mirror points. In this case, we refer to the electron as being *trapped*. We discuss trapping effects in Sect. 4.

Through the interaction of electrons with the tangled magnetic field, electrons couple to all other plasma species electromagnetically. Therefore, all these processes give rise to an anomalous transfer of momentum from electrons to ions and vice versa. In collisionless plasmas, this inter-species transfer of momentum creates *anomalous resistivity* and thus lowers the conductivity of the plasma by reducing the free streaming of electrons (Drummond and Rosenbluth 1962; Davidson and Gladd 1975; Davidson and Krall 1977; Papadopoulos 1977). This anomalous resistivity plays a crucial role, for example, in magnetic reconnection (Mozer et al. 2011; Le et al. 2018; Graham et al. 2022) and magnetogenesis (Schekochihin and Cowley 2006; Mogavero and Schekochihin 2014) in collisionless plasmas.

**Fig. 2:**
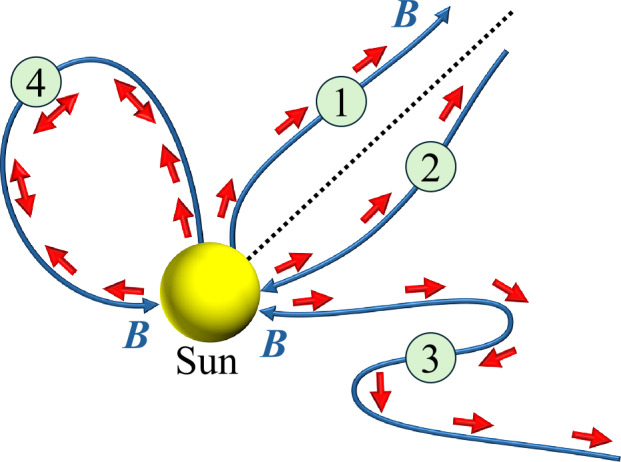
The strahl can be used as a tool to measure magnetic connectivity in the solar wind. The blue curves represent magnetic-field lines connected with the Sun. The red arrows indicate the local direction of the heat flux vector. The dashed black line represents a global separation of field-line connectivity (e.g., the heliospheric current sheet). The green circles illustrate in-situ measurement points

In space plasmas accessible to in-situ observations, we exploit the strong magnetisation of electrons to ascertain the connectivity of the magnetic field when measured by a spacecraft. In the solar wind, for instance, the majority of the electron heat flux is visible as a field-aligned beam in the electron velocity distribution function, referred to as the *strahl* (Pilipp et al. 1987; Maksimovic et al. 1997; Pierrard et al. 2001). The strahl feature originates in the solar corona and consists of magnetised electrons that move along interplanetary magnetic-field lines and away from the Sun (Owens et al. 2017). A local measurement of the strahl pitch-angle distribution thus allows us to determine the connectivity of the local field line to the solar corona (Gosling et al. 1987; Crooker et al. 2004; Macneil et al. 2020). Figure 2 illustrates this approach. At measurement point 1, the strahl propagates into the direction of the field (parallel to $\boldsymbol{B}$). The electron heat-flux vector $\boldsymbol{q}_{\mathrm {e}}$ is thus likewise parallel to $\boldsymbol{B}$. At measurement point 2, the opposite situation occurs: the strahl propagates into the direction anti-parallel to $\boldsymbol{B}$. When crossing a field-line reversal, such as at measurement point 3, the strahl propagates towards the Sun but at the same orientation with respect to $\boldsymbol{B}$ as before and after the interval when the field reversed. At measurement point 4, the electron pitch-angle distribution shows bi-directional strahl due to the connectivity to both coronal footpoints of a magnetic-field loop. The same technique is also a powerful tool for the analysis of magnetic connectivity in planetary plasma environments (e.g., Frahm et al. 2006; Tsang et al. 2015).

**Fig. 3:**
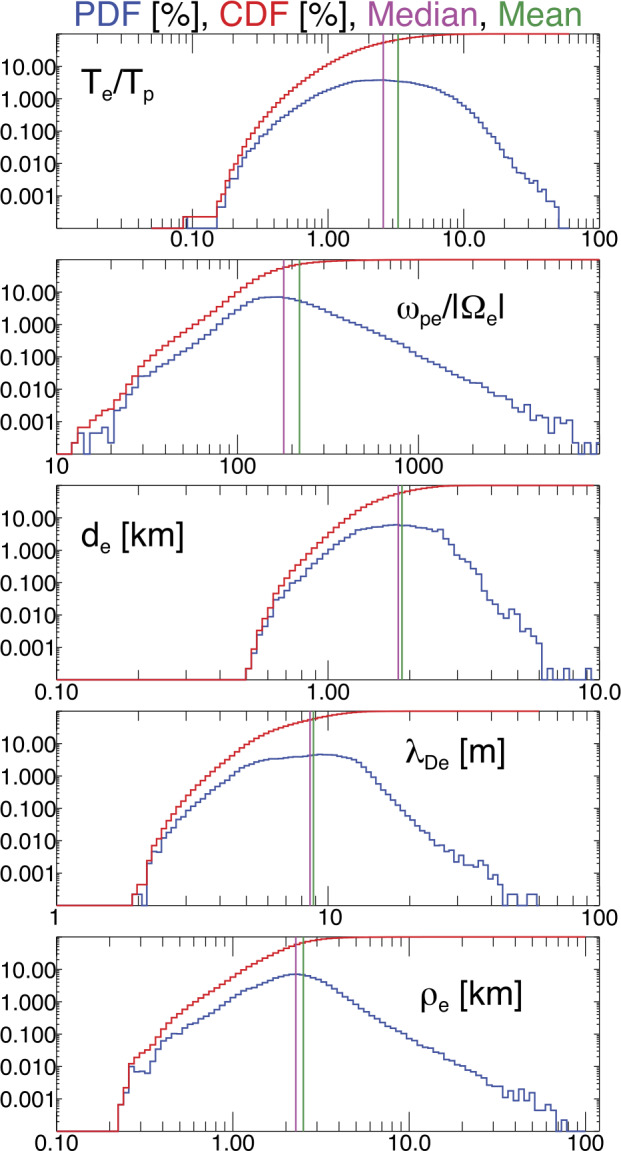
PDFs (blue histograms) and CDFs (red histograms) of several solar-wind parameters observed by Wind near 1 au. The panels (from top to bottom) are as follows: $T_{\mathrm {e}}/T_{\mathrm {p}}$, $\omega _{\mathrm{pe}}/|\Omega _{\mathrm {e}}|$, $d_{\mathrm {e}}$, $\lambda _{\mathrm{De}}$, and $\rho _{\mathrm {e}}$. For each distribution, we show the median (magenta) and mean (green) values of the data as vertical lines. The data are taken from Wilson III et al. (2023)

The solar wind provides us with a unique laboratory to study electron transport in tangled fields. It gives us access to direct in-situ measurements of electron velocity distribution functions and electromagnetic fields. At the same time, the plasma is so variable that, by sampling long statistical datasets, we have access to a broad range of plasma conditions. Figure 3 shows probability density functions (PDFs; blue histograms) and cumulative distribution functions (CDFs; red histograms) of several solar-wind parameters observed by the Wind spacecraft at a heliocentric distance of about 1 au. It is important to remember that the five plasma parameters reported in Fig. 3 are statistically correlated. Therefore, although the solar wind provides us with a wide range of plasma conditions, not all parameter combinations can be sampled with the same statistical reliability in spacecraft measurements in the solar wind at 1 au.

The top panel shows the statistical distribution of the electron-to-proton temperature ratio, $T_{\mathrm {e}}/T_{\mathrm {p}}$, indicating that on average $T_{\mathrm {e}}>T_{\mathrm {p}}$ in equatorial solar wind, which is a typical feature of slow solar wind (Salem et al. 2023). The second panel shows the ratio $\omega _{\mathrm{pe}}/|\Omega _{\mathrm {e}}|$ between the electron plasma frequency

7 $$ \omega _{\mathrm{pe}}=\sqrt{ \frac{4\pi n_{\mathrm {e}}e^{2}}{m_{\mathrm {e}}}}, $$

where $n_{\mathrm {e}}$ is the electron density, and the electron cyclotron frequency. The third panel shows the statistical distribution of the electron inertial length

8 $$ d_{\mathrm {e}}=\frac{c}{\omega _{\mathrm{pe}}}, $$

and the fourth panel shows the statistical distribution of the Debye length

9 $$ \lambda _{\mathrm{De}}=\sqrt{ \frac{k_{\mathrm {B}}T_{\mathrm {e}}}{4\pi n_{\mathrm {e}}e^{2}}}, $$

where $k_{\mathrm {B}}$ is the Boltzmann constant. The fifth panel shows the statistical distribution of the electron gyro-radius $\rho _{\mathrm {e}}$, where we evaluate $\rho _{\mathrm {e}}$ as the thermal gyro-radius, i.e., the gyro-radius of an electron with $v_{\perp}=w_{\mathrm {e}}$, where

10 $$ w_{\mathrm {e}}=\sqrt{\frac{2k_{\mathrm {B}}T_{\mathrm {e}}}{m_{\mathrm {e}}}} $$

is the thermal speed of the electrons. We note that, with these definitions, the squared ratio of the electron gyro-radius to the electron inertial length describes the ratio between the electron thermal pressure to the magnetic pressure:

11 $$ \beta _{\mathrm {e}}=\left (\frac{\rho _{\mathrm {e}}}{d_{\mathrm {e}}} \right )^{2}= \frac{8\pi n_{\mathrm {e}}k_{\mathrm {B}}T_{\mathrm {e}}}{B^{2}}. $$

The mean of $\beta _{\mathrm {e}}$ in the dataset shown in Fig. 3 is 2.31, while its median is 1.09.

The tangling of the magnetic field and the impact of tangled fields on electron transport depend on the spatial and temporal scales at which the fluctuations in the field occur. For the context of electron transport, the comparison of the spatial scales of the field’s variation with $d_{\mathrm {e}}$ and $\rho _{\mathrm {e}}$ (see also Fig. 1) provides valuable insights. For context, in the data shown in Fig. 3, the mean $d_{\mathrm {e}}$ is 1.87 km, the median $d_{\mathrm {e}}$ is 1.82 km, the mean $\lambda _{\mathrm{De}}$ is 8.84 m, the median $\lambda _{\mathrm{De}}$ is 8.58 m, the mean $\rho _{\mathrm {e}}$ is 2.51 km, and the median $\rho _{\mathrm {e}}$ is 2.28 km. Figure 3 also highlights that the near-Earth solar wind exhibits a large separation of the characteristic electron scales, which poses big challenges when attempting to model this system numerically with kinetic simulations (e.g., see Wilson III et al. 2021). The median value of $\omega _{\mathrm{pe}}/|\Omega _{\mathrm{e}}|$ is nearly 200.

Unlike in the solar wind, other examples of field tangling in astrophysics, such as solar-flare turbulence, cannot be measured in situ, and so must be inferred from remote-sensing observations. In solar physics, we often use spectral-line observations from instrumentation like the Hinode EUV Imaging Spectrometer (EIS; Culhane et al. 2007), Interface Region Imaging Spectrometer (IRIS; De Pontieu et al. 2014), and upcoming missions such as the MUlti-slit Solar Explorer (MUSE; De Pontieu et al. 2022) to infer field tangling and its impact on electron transport.

This article reviews a selection of transport phenomena relating to electrons in tangled magnetic fields. It focuses on collisionless space and astrophysical plasmas, in which collective behaviour dominates the interactions between electrons and variations in the magnetic field across scales. We first introduce contemporary ideas for the creation of tangled magnetic fields. Magnetohydrodynamic (MHD) turbulence and fluid instabilities tangle magnetic fields and thus naturally create inhomogeneities in the field (Sect. 2.1). On smaller scales, kinetic instabilities tangle the magnetic field (Sect. 2.2). In this context, we also briefly point at the magnetogenesis problem and the turbulent dynamo, which relate to the creation of tangled magnetic fields from an unmagnetised initial state. Through the tension force and the exchange of magnetic stresses, however, magnetic fields resist being tangled (Sect. 2.3). We briefly discuss the modulation of field-parallel heat flux by kinetic instabilities in Sect. 3. A key aspect of electron transport in tangled fields relates to trapping effects in inhomogeneous magnetic-field configurations (Sect. 4), for which planetary magnetospheres provide many important example cases. We also discuss de-trapping effects due to the breaking of adiabaticity of the electron trajectories (Sect. 4.4). Electron diffusion and energisation across the magnetic field are the scope of Sect. 5, which discusses the phenomenology of electron scattering and then presents an overview over diffusion in energy and space. The article concludes with a brief summary and outlook in Sect. 6.

## The Creation of Tangled Magnetic Fields

In this section, we discuss the creation of tangled fields by two broad classes of processes: those associated with fluid instabilities and turbulence (Sect. 2.1), and those generated by the conversion of energy from particles to fields through kinetic processes that disrupt particle transport (Sect. 2.2).

### Field Tangling by Turbulence and Fluid Instabilities

Turbulence is present in most space and astrophysical plasmas: the solar corona (Cranmer and Winebarger 2019), the solar wind (Bruno and Carbone 2013), the interstellar medium (Armstrong et al. 1995; Ferrière 2020; Fraternale and Pogorelov 2021), and the intracluster medium (Fabian et al. 2006; Vazza et al. 2012; Cho et al. 2022). In this section, we discuss the turbulent cascade, its multi-scale structure, various types of anisotropies of turbulent fluctuations, and the role of fluid instabilities.

**Fig. 4:**
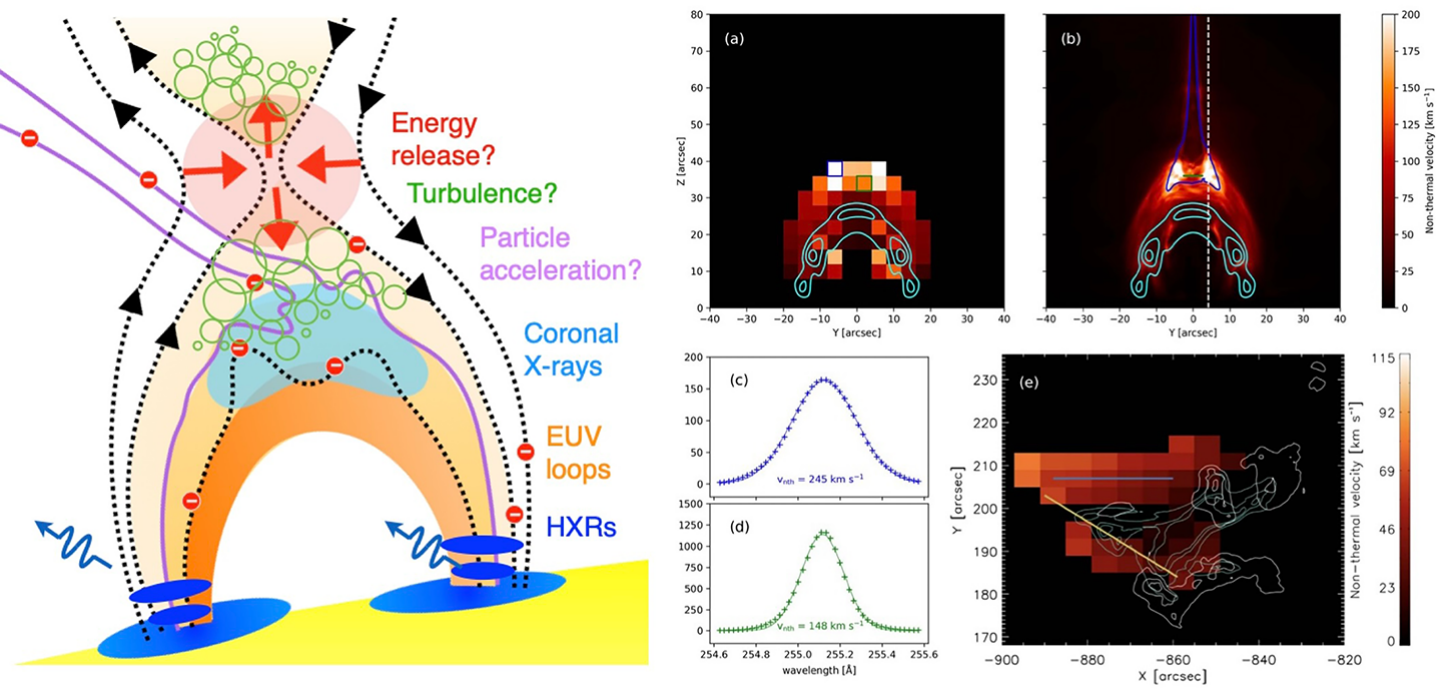
Left: Simple cartoon depicting the standard flare model showing possible sites of energy release, generation of turbulence in the corona, tangled fields, particle acceleration, heating, and subsequent X-ray emissions. Right: Flare observation of extended regions of turbulence in the solar corona (panel e), inferred from the presence of excess spectral-line broadening (Stores et al. 2023). The observation is compared with extended regions of turbulence generated in MHD simulations of the KHI (panels a through d). Figure taken from Ruan et al. (2023)

Solar flares are one example plasma process in which field tangling by turbulence plays a crucial role, especially for the transport of electrons. Solar flares change dynamically in space and time during their evolution, possibly favouring stochastic acceleration processes (e.g., Larosa and Moore 1993; Petrosian 2012; Riquelme et al. 2022) generated by turbulence and plasma waves. Irrespective of the exact acceleration mechanism(s), turbulence is likely to play a key role. For instance, shocks require repeated particle crossings for efficient acceleration, possibly being fed by turbulence. Fluid instabilities, such as the *tearing-mode instability* with the generation of magnetic islands and the *Kelvin–Helmholtz instability* (KHI) created in the plasma sheet above coronal loop tops can generate turbulence. Moreover, ample evidence for turbulence comes from spectral-line observations, specifically broadening, and possibly from spectral-line shapes (e.g., Jeffrey et al. 2017). Spectral lines are often wider than expected from the underlying ion thermal motions alone, leading to *non-thermal line broadening* (Milligan 2015). This excess broadening is often attributed to random macroscopic plasma motions and turbulence. Observations show that turbulence may play a role throughout the flare from coronal reconnection sites to the lower chromosphere. Spectral observations (Doschek et al. 2014; Stores et al. 2021) show distinct line-broadening patterns, such as non-thermal line broadening increasing with temperature and height in a coronal loop, or a decrease in line broadening as we move from the loop top down the loop legs (see Fig. 4). These observations suggest that, while turbulence may be most crucial in the loop tops, it has extended presence in multiple locations during a flare and plays a key role in energy transfer throughout the event. In addition, spectral-line broadening occurs also within the transition region and chromosphere, even during the flare rise phase, giving a hint at the presence of turbulence (Milligan 2011; Jeffrey et al. 2018). Other studies have shown non-thermal broadening in active regions before the flare (Harra et al. 2013) and also at later times in long-duration flares (French et al. 2019). Magnetic energy must transfer from large to small scales in flares, and turbulence is an important mechanism for such a transfer of energy (see Sect. 2.1.1). A substantial fraction of the released energy goes into the flare-acceleration of electrons with estimates of 10-50% (Emslie et al. 2012). Multi-wavelength observations allow us to estimate the partitioning of energy in flares (Kontar et al. 2017). In this study, X-ray observations of the spectral-line broadening suggest that the instantaneous kinetic energy associated with turbulence is sufficient to power the acceleration of non-thermal electrons. Moreover, the estimation of the timescale for dissipation from turbulent kinetic energy to electron thermal energy is of order 1-10 seconds, similar to predictions based on MHD modelling (Kontar et al. 2017).

#### The Turbulent Cascade and Multi-Scale Structure

A flow is turbulent when the nonlinear terms in the dynamical equations describing the system – for example, the Navier–Stokes equation in hydrodynamics, the MHD equations for collisional magnetofluids, or the Vlasov–Maxwell set of equations for collisionless plasmas – are dominant and lead to a transfer of energy across scales. When the timescale associated with these nonlinear terms is comparable to or shorter than the timescales associated with linear processes, the system develops into a stochastic state (Sreenivasan 1999; Goldreich and Sridhar 1995). A state of homogeneous and statistically stationary turbulence emerges when the correlation length $\ell _{\mathrm {c}}$ and the correlation time $\tau _{\mathrm {c}}$ of the auto-correlation functions of the characteristic quantities of the system are small compared to the system size and its global evolution time (Matthaeus and Goldstein 1982). The nonlinear terms in the dynamic equations for these turbulent systems transfer energy across scales, which ultimately leads to dissipation at small scales (Bruno and Carbone 2013; Matthaeus et al. 2015; Marino and Sorriso-Valvo 2023). In the classic picture of fluid turbulence (Kolmogorov 1941), this transfer begins at the driving scale and ends at the scales associated with collisional dissipation. The driving scale, on which the system is unstable, is comparable to $\ell _{\mathrm {c}}$ and thus a large scale compared to the dissipation scales. The fluctuations then become nonlinear and develop a *turbulent cascade* that proceeds towards smaller scales.

In magnetofluids, which evolve according to the MHD equations, the magnetic field $\boldsymbol{B}$ is coupled to the flow through the magnetofluid’s bulk velocity $\boldsymbol{U}$ according to the induction equation

12 $$ \frac{\partial \boldsymbol{B}}{\partial t}= \text{{$\boldsymbol{{\nabla }}$}}\,\text{{$\boldsymbol{{\times }}$}}\,\left ( \boldsymbol{U}\,\text{{$\boldsymbol{{\times }}$}}\,\boldsymbol{B}\right )+ \eta \nabla ^{2}\boldsymbol{B}, $$

where $\eta $ is the magnetic diffusivity. The flow is affected by the magnetic field through the Lorentz force in the MHD momentum equation. Under ideal conditions (i.e., $\eta \rightarrow 0$), the field is *frozen into the flow* (Alfvén 1943). Therefore, when the flow develops turbulent fluctuations, so does the magnetic field. In this way, MHD turbulence creates tangled fields across a range of spatial and temporal scales. These in turn affect the trajectories of electrons.

Once the fluctuations in the system are nonlinear and cascade, they generate and couple to fluctuations on different scales. The kinetic energy of the flow is an ideal invariant that cascades conservatively in hydrodynamic turbulence within the so-called *inertial range* of scales, which is the range of scales between driving and dissipative scales. In three-dimensional incompressible MHD turbulence, the total energy, the cross-helicity, and the magnetic helicity are the three cascading invariants (Matthaeus and Goldstein 1982).

**Fig. 5:**
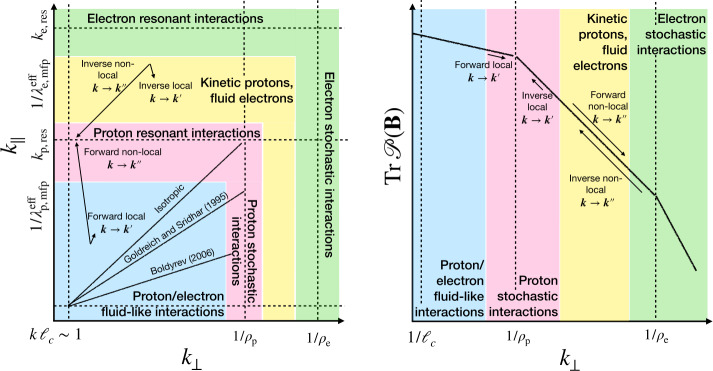
Scale-dependent fluctuations in the magnetic field and their interactions with protons and electrons through the turbulent cascade, wave–particle resonances, and stochastic interactions. The left panel shows a two-dimensional plane of wavevector space with the field-perpendicular component $k_{\perp}$ on the horizontal and the field-parallel component $k_{\parallel}$ on the vertical axis. The non-arrowed solid lines represent theoretical predictions for the cascade of MHD turbulence. The right panel shows the trace power spectral density $\mathcal {P}(\boldsymbol{B})$ of the magnetic field integrated over $k_{\parallel}$. The colours indicate different regimes discussed in the text. Arrows illustrate forward and inverse spectral transfer which can be local ($\boldsymbol{k} \rightarrow \boldsymbol{k}^{\prime}$) or non-local ($\boldsymbol{k} \rightarrow \boldsymbol{k}^{\prime \prime}$) in wavevector space. At scales above the effective mean free paths $\lambda _{\mathrm{p, mfp}}^{\mathrm{eff}}$ and $\lambda _{\mathrm{e, mfp}}^{\mathrm{eff}}$, collisionless processes fluidise protons (blue regime) or electrons (yellow regime), respectively. Resonant interactions dominate at $k_{\parallel}\sim k_{j,\mathrm{res}}$ for species $j$

The power spectral densities of the bulk velocity and magnetic field in MHD turbulence follow power laws in the inertial range, which is also observed in the solar wind (Coleman 1968; Bruno and Carbone 2013). The power spectral density as a function of wavenumber measures the distribution of the signal’s power over spatial scales. For a generic signal $f(r)$, the power spectral density is $S(k)= \mathcal {F}R(k)$, where $\mathcal {F}R(k)$ is the Fourier transform of the spatial auto-correlation function $R(\rho )=\langle f(r)f(r+\rho ) \rangle _{r}$, where the angle brackets indicate averaging over $r$. The Fourier transform translates the $r$-dependence of the signal $f$ into a dependence on wavenumber $k$. This concept can be easily extended to multi-dimensional signals that depend on all three spatial coordinates $\boldsymbol{r}$. A schematic of a typical power spectrum is shown in Fig. 5. The panel on the right-hand side shows that the fluctuation amplitude decreases with decreasing scale.

Multiple models exist for the description of the cascade of MHD turbulence. In Fig. 5, we indicate three representative models through black solid lines: an isotropic cascade, the critical-balance model (Goldreich and Sridhar 1995), and the dynamic-alignment model (Boldyrev 2006). The distribution of energy through the MHD cascade is a matter of ongoing research (Chandran et al. 2015; Mallet et al. 2015; Schekochihin 2022).

The large scales of the system, typically referred to as the *driving scales*, provide large-amplitude fluctuations that drive the turbulent evolution of the system (shown as the scale with $k \ell _{\mathrm {c}} \sim 1$ in Fig. 5). In the case of the solar corona and solar wind, the reflection of Alfvén waves propagating away from the Sun plays a key role in the development of turbulence (Heinemann and Olbert 1980; Chandran and Hollweg 2009; Bruno and Carbone 2013; Cranmer and Winebarger 2019; Chandran and Perez 2019). These Alfvén waves are launched by the footpoint motion of the magnetic-field lines in the photosphere and lower corona. When these waves propagate into gradients in the coronal background field strength and density, they undergo partial reflection that generates Sunward-propagating Alfvén waves. Oppositely propagating Alfvén waves interact nonlinearly, and the system becomes turbulent (Drake et al. 2013; Nielson et al. 2013; Pezzi et al. 2017; Verniero et al. 2018). A combination of models, simulations, and (indirect) observations provides good evidence for the wave-reflection model in the corona and near-Sun region (Cranmer et al. 2007; Cranmer and Winebarger 2019). Moreover, the parametric decay of large-amplitude Alfvén waves, in which a wave decays into two oppositely propagating Alfvén waves and a compressive wave, leads to counter-propagating waves in the solar wind (Goldstein 1978; Tenerani et al. 2017). The observation that the solar wind is turbulent throughout most of the heliosphere suggests that the driving of turbulence must persist as the solar wind propagates away from the Sun (Zank et al. 1996; Smith et al. 2006). At large distances from the Sun, the driving of large-scale fluctuations by interstellar pick-up ions injects fresh energy into the turbulent cascade (Breech et al. 2008).

At large scales, the system is in a fluid-like regime that is appropriately described through a low-order set of velocity moments of the kinetic equation (Verscharen et al. 2017). Such a description is justified when the system can be approximated with a simple equation of state or when the evolution of the system does not rely on higher-order moments altogether (e.g., in non-compressive Alfvénic turbulence or in MHD turbulence). In collisionless plasmas, however, a low-order truncation of the velocity-moment hierarchy is not valid, and more complex kinetic models are required to capture the evolution accurately. In collisional plasmas, this transition to the kinetic regime occurs when fluctuations have smaller scales than the collisional mean free path of species $j$ (i.e., at scales with $k\lambda _{j, \mathrm{mfp}}\gtrsim 1$). However, Coulomb collisions are not sufficient to explain the observed fluidisation in collisionless heliospheric and astrophysical plasmas at large scales. Therefore, other processes than collisions must be responsible for the observed fluidisation of these systems at large scales. We associate these processes with *effective mean free paths* in analogy to the collisional fluidisation (Coburn et al. 2022). We define $\lambda _{\mathrm{p, mfp}}^{\mathrm{eff}}$ and $\lambda _{\mathrm{e, mfp}}^{\mathrm{eff}}$ for protons and electrons, respectively. The transition from the fluid into the kinetic regime then occurs when $k_{\parallel} \lambda _{\mathrm{p, mfp}}^{\mathrm{eff}} \gtrsim 1$ for protons and when $k_{\parallel}\lambda _{\mathrm{e, mfp}}^{\mathrm{eff}} \gtrsim 1$ for electrons. In Fig. 5, we highlight the relevant transitions and the associated regimes.

Other important scales of the system are those at which thermal particles become resonant with the fields and fluctuations of the fields perturb the particle gyro-radii, causing their trajectories to develop into a stochastic state. Resonant interactions require that particles and wave fields fulfil a resonance condition in which the field-parallel wavevector component $k_{\parallel}$ matches a resonance wavenumber $k_{j,\mathrm{res}}$ for particle species $j$. This resonance wavenumber depends on $v_{\parallel}$ of the resonant particles, the real part $\omega _{\mathrm {r}}$ of the wave frequency of the resonant waves at wavenumber $k_{\parallel}=k_{j,\mathrm{res}}$, and potentially the cyclotron frequency of the resonant particles through the resonance condition

13 $$ \omega _{\mathrm {r}}(k_{j,\mathrm{res}})=k_{j,\mathrm{res}}v_{ \parallel}+n\Omega _{j}, $$

where $n$ is an integer that classifies the order of the resonance (see also Sect. 4.4.1). Stochastic interactions require that the wavevector component $k_{\perp}$ perpendicular to the background magnetic field is comparable to $1/\rho _{j}$ for species $j$. Both resonant and stochastic processes can act on thermal particles or on energetic particles of species $j$ depending on the relevant $k_{j,\mathrm{res}}$ and $\rho _{j}$. The scales for resonant and stochastic interactions are highlighted in pink (protons) and green (electrons) in Fig. 5. The ordering of scales in Fig. 5 follows the ordering for thermal particles in typical solar wind at 1 au (Coburn et al. 2022) but other orderings are possible in different environments or for other particle species.

Weakly collisional systems not only exhibit significant fluctuations in higher-order moments of the velocity distributions but also often develop non-equilibrium features in the background distributions of the particles that drive instabilities and thereby modify the field geometry. Unlike a turbulent cascade, these instabilities create non-local spectral transfer of fluctuation energy. In Fig. 5, we indicate schematically non-local direct interactions that arise, e.g., when large-scale fluctuations cause the system to become kinetically unstable by generating temperature anisotropy (cf. Verscharen et al. 2016; Arzamasskiy et al. 2023). The instability then leads to the growth of fluctuations at the proton gyro-scale. We discuss these processes further in Sect. 2.2. Magnetic reconnection, in turn, can lead to non-local inverse transfer of energy (cf. Franci et al. 2017; Zhou et al. 2022, 2024), for example, through the coalescence of magnetic islands formed by reconnection.

#### Anisotropies of the Turbulent Fluctuations

The geometric structure of the tangled fields created by turbulence is not isotropic (Strauss 1976; Montgomery and Turner 1981, 1982; Shebalin et al. 1983; Higdon 1984; Goldreich and Sridhar 1995, 1997; Horbury et al. 2008; Bruno and Carbone 2013). In fact, there are multiple aspects of the turbulent fluctuations that are anisotropic with respect to the background magnetic field (Horbury et al. 2012). The components of the fluctuating field amplitude in the direction perpendicular to the background field are typically greater than the component of the fluctuating field amplitude parallel to the background field (*variance anisotropy*). The energy transfer rate depends on the direction of the wavevector and is thus anisotropic (*cascade anisotropy*). Smaller-scale fluctuations are typically more elongated along the background field than larger-scale fluctuations, resulting in an anisotropy in the distribution of power across wavevector space (*wavevector anisotropy*). Lastly, the spectral index depends on the direction of the wavevector (*spectral-index anisotropy*). The anisotropies as well as the overall power levels and polarization properties of the turbulence vary over time and are thus likely not universal (see, e.g., Schekochihin et al. 2009; Oughton and Matthaeus 2020; Schekochihin 2022; Howes 2024). The anisotropy of the fluctuations has drastic implications for the field’s ability to affect electron transport. Therefore, the generation of field structures and the nature of the cascade are integral parts of our understanding of tangled magnetic fields in plasmas.

#### Fluid Instabilities

Fluid instabilities tangle the magnetic field on length scales much greater than the thermal electron scales. Therefore, electrons usually remain magnetised in these structures and follow the field lines. However, fluid instabilities can excite a turbulent cascade, bringing fluctuations to smaller scales through nonlinear interactions. In addition, energetic electrons, such as cosmic-ray electrons, may have gyro-radii of order the size of the structures created by fluid instabilities. In those cases, large-scale structures may lead to diffusion or trapping of energetic electrons.

Fluid instabilities represent the growth of perturbations in (magneto-)hydrodynamic systems. The MHD description applies on scales associated with the driving and inertial range of the turbulence (scales in the blue shaded region of Fig. 5). A key instability in space and astrophysical plasmas is the KHI, which arises when there is a shear in a fluid flow (Helmholtz 1868; Kelvin 1871; Rayleigh 1880; Chandrasekhar 1961). In a magnetised fluid, the KHI naturally produces tangled fields in its nonlinear phase as it generates vortices and rolls in the fluid and the frozen-in magnetic field, ultimately leading to plasma mixing as well as mass and energy transport (Nykyri and Otto 2001; Hasegawa et al. 2004; Matsumoto and Hoshino 2006; Henri et al. 2013; Faganello and Califano 2017). An easily accessible location for the in-situ observation of the KHI is in the shear flows at the flanks of Earth’s magnetopause, which is the interface between the magnetosheath and the magnetosphere (Hasegawa et al. 2004, 2006; Foullon et al. 2008; Taylor et al. 2012; Eriksson et al. 2016; Blasl et al. 2022). The KHI is often observed at large (fluid) scales where it acts as an additional driver for a turbulent cascade. At the interface between Kelvin–Helmholtz vortices, ion-scale regions of intense magnetic stresses are observed (Sorriso-Valvo et al. 2019). On the smaller electron scales, when the ions and electrons decouple, electron shears can lead to electron KHI (Fermo et al. 2012; Zhong et al. 2018; Che and Zank 2023). Its nonlinear phase generates tangling of field lines on electron scales and structures such as plasmoids and flux ropes.

In accretion discs, the *magnetorotational instability* (MRI) is a linear instability that facilitates angular-momentum transport and mass accretion in a wide range of astrophysical discs (Balbus and Hawley 1991; Hawley and Balbus 1991; Balbus and Hawley 1998). It arises when a weak magnetic field threads a rotating disc in which the angular velocity decreases with radius, such as in accretion discs around stars or black holes. By exerting torques on magnetically tethered fluid elements, the perturbed magnetic field acts in such a way that outwardly (inwardly) displaced fluid elements gain (lose) angular momentum as they continue to move outwards (inwards). In this way, the free energy of the disc’s differential rotation is converted into radial and azimuthal motions, ultimately enabling mass accretion onto the central massive object. More than three decades of numerical simulations have demonstrated that the nonlinear evolution of the MRI is a state of turbulence in which spatial anisotropy on large scales is structured by the differential rotation of the disc. More recent simulations reveal what appears to be an inertial-range power spectrum approaching that of Alfvénic guide-field turbulence at small scales (Walker et al. 2016; Kawazura and Kimura 2024). The MRI is expected to play a critical role in many astrophysical systems, including protoplanetary discs, where it is believed to influence gas dynamics and planet formation efficiency (Mohanty et al. 2018; Jankovic et al. 2021). It also plays an important role in discs around compact objects like neutron stars and black holes, powering high-energy emission through efficient angular-momentum transport (Kiuchi et al. 2015). In galactic discs, the MRI may also contribute to the generation of large-scale magnetic fields (Machida et al. 2013).

Heat transport itself can create tangled magnetic fields through buoyancy instabilities, provided that the transport occurs predominantly along magnetic-field lines. One of these instabilities is the *magnetothermal instability* (MTI; Balbus 2000, 2001). It arises when the plasma temperature gradient is parallel to the gravitational force (i.e., hot at the bottom, cold at the top) and leads to strongly tangled magnetic-field configurations (McCourt et al. 2011). The conditions for the driving of the MTI occur in the outskirts of galaxy clusters (e.g., Kempf et al. 2023) and in hot, dilute accretion flows (Bu et al. 2011). However, the action of the MTI in weakly collisional plasmas depends critically on the plasma viscosity (Kunz 2011; Kunz et al. 2012) and on microphysical wave–particle interactions that affect heat transport (Xu and Kunz 2016; Perrone et al. 2024).

Of similar relevance to the creation of tangled fields is the *heat-flux buoyancy-driven instability* (HBI; Quataert 2008). It acts when the temperature gradient is anti-parallel to the gravitational force (i.e., cold at the bottom, hot at the top), as is the case in the centres of cool-core galaxy clusters. This instability tends to re-arrange magnetic fields into a tangential configuration, suppressing field-aligned heat transport between different radii (Parrish et al. 2009; Bogdanović et al. 2009). However, it too depends critically on the plasma viscosity (Kunz 2011; Kunz et al. 2012), as well as on the amount of background turbulence driven by, e.g., feedback from the cluster’s central dominant galaxy (Ruszkowski and Oh 2010).

### Field Tangling by Kinetic Instabilities

In magnetised plasmas, kinetic instabilities create fluctuations in the magnetic field near the ion or electron gyro-radius scales, which may affect the transport of electrons. In Fig. 5, the creation of these fluctuations is indicated as non-local power transfer to the scales at which resonant or stochastic interactions take place. Kinetic instabilities can modulate the magnetic-field direction and amplitude at electron (and ion) scales, lead to the creation of turbulent fields through magnetogenesis followed by a turbulent dynamo process, and create large-scale fluctuations in the magnetic field when driven by energetic particles.

#### Ion-Scale and Electron-Scale Instabilities in Magnetised Plasma

Many plasma instabilities on ion or electron scales feed off pressure anisotropy as their main source of free energy. The generation of pressure anisotropy is a generic process in any magnetised, weakly collisional, and dynamic plasma system (see also Sect. 2.2.2).

Pressure anisotropy with respect to the background magnetic field can drive gyro-scale instabilities such as the *mirror-mode* (Hasegawa 1969; Southwood and Kivelson 1993), *firehose* (Chandrasekhar et al. 1958; Rosenbluth 1958; Parker 1958a; Vedenov and Sagdeev 1961; Quest and Shapiro 1996; Gary et al. 1998), *ion-cyclotron* (Kennel and Petschek 1966; Gary 1992), and *whistler-wave instabilities* (Kennel and Petschek 1966; Gary and Wang 1996). These instabilities serve to limit the pressure anisotropy of the particle velocity distribution functions by creating ion-scale or electron-scale fluctuations that, in their nonlinear phase, trap and/or scatter particles in pitch-angle. Although these instabilities do not produce strongly tangled magnetic fields per se, the mirror-mode instability can give rise to ion-gyro-scale fluctuations with $\delta B/B \sim 0.3$ (Kunz et al. 2014; Riquelme et al. 2015). These fluctuations are capable of generating magnetic holes in which electrons can be trapped, affecting the transport of electrons (Komarov et al. 2016; Riquelme et al. 2016; Liu et al. 2025) and modifying the electron velocity distribution within the holes (Jiang et al. 2022; Ley et al. 2024). Section 4.2 discusses these trapping effects in more detail.

Many ion-scale and electron-scale instabilities are also associated with collisionless shock waves, and indeed, many strong electron-scale fluctuations are commonly observed near shock waves. A collisionless plasma shock is most often a nonlinearly steepened fast-magnetosonic/whistler wave that has reached a balance between steepening and dispersion. The resultant discontinuity continually generates free energy from the driver of the shock. Since these shocks are a steepened form of a dispersive wave mode, the shock itself can radiate waves on the same branch of the dispersion relation (Tidman and Northrop 1968; Krasnoselskikh et al. 2002), known as *whistler precursors*. Whistler precursors can also be generated by shock-reflected particles through a modified *two-stream instability* (Matsukiyo and Scholer 2006). These waves have wavelengths that span from above ion scales to electron scales (Hull et al. 2012, 2020; Wilson III et al. 2012, 2013, 2014a,b, 2017). Whistler precursors can also greatly affect the incident flow (both ions and electrons), and within these flow modulations, even smaller-scale electrostatic waves can be generated (Wilson III et al. 2007, 2014a,b, 2021). The same free-energy source that generates whistler precursors at strong shocks (i.e., through particle reflection) can generate much smaller-scale waves as well. Waves radiated by *electron-cyclotron drift instability* (ECDI) at shocks (e.g., Breneman et al. 2013; Wilson III et al. 2010, 2014b) are direct evidence of electron–ion coupling since the relative drift between reflected ions and incident electrons acts as their free-energy source. The waves heat suprathermal electrons perpendicular to the quasi-static magnetic field, thermal electrons parallel to the field, and generate parallel ion tails through the coupling between Doppler-shifted ion-acoustic modes and electron Bernstein modes (Amano et al. 2026).

#### Magnetogenesis and the Turbulent Dynamo

The tangling of magnetic fields is often related to two scientific challenges: (1) the origins of magnetic fields in the first place, and (2) the means by which these seed fields are amplified to dynamical strengths. The interplay between kinetic instabilities and turbulence forms a possible scenario to explain this chain of events that leads to tangled magnetic fields.

When considering the first challenge, the *Weibel instability* (Weibel 1959) is a promising candidate mechanism to provide a seed magnetic field. This instability is driven by anisotropic particle pressure configurations in (nearly) unmagnetised plasmas. Small magnetic-field fluctuations with wavevectors oriented in the direction of the colder part of the velocity distribution reinforce fluctuations in the current density oriented in the direction of the hotter part of the distribution. Once unstable, the plasma grows fluctuations in the magnetic field until the field becomes strong enough to magnetize the plasma and thereby affect the trajectories of the electrons. Additional trigger mechanisms may be in place, for instance two-stream instabilities that create Langmuir waves due to the differential streaming of electrons and ions in regions with spatially modulated particle densities (Schlickeiser and Shukla 2003). Resonant wave–particle interactions relax the two-stream distributions to be anisotropic, which then triggers a secondary Weibel instability. An initially imposed velocity shear can also generate electron anisotropy, which then drives the Weibel instability (Pucci et al. 2021; Zhou et al. 2022).

After the creation of the seed field through the Weibel instability, a turbulent dynamo process sets in that leads to the further growth of the magnetic field, until the field growth is balanced with dissipation, for instance through magnetic reconnection (Sironi et al. 2023). This proposed dynamo process addresses the second challenge of magnetogenesis: the growing of the seed field into a dynamically important large-scale magnetic field.

**Fig. 6:**
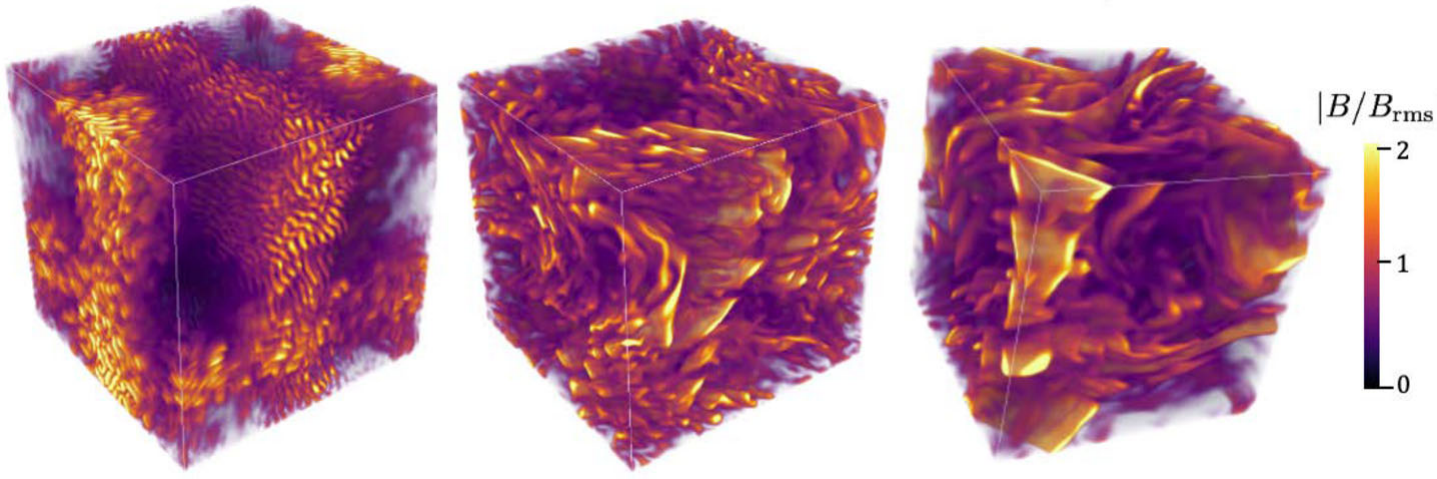
Magnetogenesis through the Weibel instability and the turbulent dynamo. The three panels show snapshots from a kinetic particle-in-cell simulation. The colour indicates the magnitude of the magnetic field normalised to its root-mean-square value. Left: at the time of peak growth of the Weibel instability. Middle: after one large-scale turnover time. Right: saturated state of the dynamo. From Zhou et al. (2024)

Figure 6 demonstrates the involved stages in a fully kinetic particle-in-cell simulation (Zhou et al. 2024). The left panel shows the simulation domain at the time when the Weibel instability is at its peak growth, and the growing kinetic-scale fluctuations are apparent. The field growth saturates as the fluctuations disrupt the electron gyro-motion. Next, the filamentary field coalesces, and the saturated field is transported to larger scales through magnetic reconnection. The result of this process is visible in the middle panel of Fig. 6, which shows a snapshot of the simulation domain after one turbulent turnover time. The system then transitions to the dynamo saturation stage, shown in the right panel of Fig. 6.

**Fig. 7:**
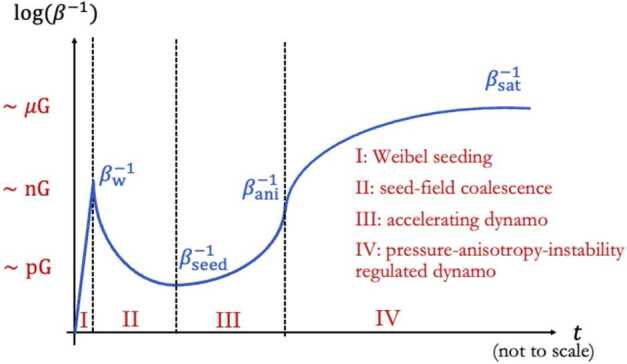
The magnetogensis scenario proposed by Zhou et al. (2024). The red labels on the vertical axis indicate typical field strengths in astrophysical plasmas during the evolutionary phases of this scenario, and $\beta $ is the ratio between the thermal and magnetic pressure. Phase I describes the creation of the seed field through the Weibel instability. In Phase II, the field undergoes coalescence and magnetic reconnection (cf. Zhou et al. 2022). In Phases III and IV, the plasma is in a collisionless dynamo state, which is ultimately regulated by pressure-anisotropy instabilities (Rincon et al. 2016; St-Onge and Kunz 2018). From Zhou et al. (2024)

Figure 7 depicts the time evolution of the phases (from Phase I to Phase IV) proposed in this magnetogenesis scenario. Future simulations must seek to connect consistently the seed and growth problems of magnetogenesis by fully realising a simulation that begins in Phase I and ends in Phase IV. At the end of Phase III, the large-scale fluctuations of the magnetised plasma trigger mirror-mode and firehose instabilities, which self-regulate the pressure anisotropy in the plasma at later stages. This self-regulation acts like an effective viscosity that controls the parallel rate of strain of the plasma and the rate of magnetic-field amplification (Rincon et al. 2016; St-Onge and Kunz 2018). A dynamo in a collisionless plasma with a field-strength-dependent effective collisionality can lead to the rapid growth of the seed magnetic field (Schekochihin and Cowley 2006; Mogavero and Schekochihin 2014). At all stages of this proposed scenario for magnetogenesis, tangled fields occur and play a crucial role for the transport of electrons.

#### Field Generation by Energetic Particles

In addition to field generation by thermal particles, also energetic particles can drive instabilities that generate and modulate magnetic fields. For instance, cosmic rays escaping from their sources along the local magnetic field can drive a variety of *streaming instabilities*, including two resonant types: *the gyro-resonant instability* (Kulsrud and Pearce 1969; Lebiga et al. 2018; Holcomb and Spitkovsky 2019; Bai et al. 2019; Bambic et al. 2021; Plotnikov et al. 2021) and the *intermediate-scale instability* (Shalaby et al. 2021, 2023; Lemmerz et al. 2025). These instabilities influence cosmic-ray transport in the interstellar medium, the intracluster medium, and near cosmic-ray sources such as supernova remnants.

Resonant streaming instabilities result in small magnetic-field amplifications with $\delta B/B \ll 1$ in environments like the interstellar and intracluster media (Holcomb and Spitkovsky 2019). However, near cosmic-ray sources such as upstream of non-relativistic shocks, magnetic amplification by resonant streaming instabilities can reach $\delta B/B \sim 1$ due to the high energy density of the streaming cosmic rays in these environments, as shown by kinetic shock simulations (Caprioli and Spitkovsky 2014a).

Upstream of shocks with sufficiently high Mach numbers, a different branch of the streaming instability, the *non-resonant streaming instability*, dominates (Bell 2004; Amato and Blasi 2009; Matthews et al. 2017). In this regime, the magnetic-field amplification reaches $\delta B/B \gg 1$ as shown in simulations (Caprioli and Spitkovsky 2014a). In the presence of the large energy density of the streaming cosmic rays, the instability grows on scales much smaller than the cosmic-ray gyro-radius, and thus the amplified field has a minimal impact on the particle trajectories. Consequently, the electric current of the cosmic rays, which drives the instability, remains weakly affected by the growing fluctuations, allowing for continued growth and thus eventually large field amplifications (e.g., Riquelme and Spitkovsky 2009).

As the growing modes become nonlinear, the cosmic-ray gyro-radius decreases due to the increasing field strength while the non-resonant instability transitions to larger scales at which large-scale instabilities may further contribute to tangle the magnetic field. This change eventually affects the cosmic-ray trajectories, producing saturation when the magnetic power is concentrated at scales near the cosmic-ray gyro-radius.

The non-resonant streaming instability occurs for any orientation of the cosmic-ray current with respect to the background magnetic field (Bell 2005). In particular, the case in which the cosmic-ray current is perpendicular to the background field has the potential to generate highly tangled fields (Riquelme and Spitkovsky 2010). Due to its large field amplification, the non-resonant streaming instability can significantly reduce the diffusion coefficients of both energetic ions and energetic electrons in the upstream medium of non-relativistic shocks, impacting the maximum energy of shock-accelerated particles (Reville and Bell 2012; Caprioli and Spitkovsky 2014b) as well as their spectra (Caprioli et al. 2020; Cristofari et al. 2021; Diesing and Caprioli 2021).

Cosmic-ray pressure effects may cause the inflation of large bubble-like structures near cosmic-ray sources as potential sites of high-intensity gamma-ray emission (Schroer et al. 2021, 2022).

### The Magnetic Field’s Resistance to Being Tangled

Magnetic fields, once generated, resist being tangled. In MHD, the magnetic tension force ${\propto}(\boldsymbol{B}\,\text{{$\boldsymbol{{\cdot }}$}}\, \text{{$\boldsymbol{{\nabla }}$}})\boldsymbol{B}$ accelerates the conducting fluid, and thus the frozen-in magnetic-field lines, so as to straighten the field. One consequence of this effect for rotating, axisymmetric, ideal MHD systems is captured by *Ferraro’s isorotation theorem*. It states that the angular velocity of a conducting fluid must be constant along magnetic-field lines in steady state (Ferraro 1937). As an application of the theorem, magnetospheres with poloidal magnetic fields around planets and stars co-rotate with their central bodies when in equilibrium. A poloidal field cannot exist in equilibrium without co-rotation because the frozen-in plasma would wind up the magnetic field in this case and build up magnetic tension. It would create toroidal field out of the initial poloidal dipole field by advection (Ogilvie 2016).

In the case of a planetary magnetosphere, the ionosphere co-rotates with the planet due to collisional friction with the atmosphere. As per Ferraro’s isorotation theorem, the co-rotating plasma of the ionosphere would stretch the magnetic field and create a time-dependent state of the field in the magnetosphere. Instead, if the Alfvén-wave propagation timescale is smaller than the dynamical timescale, electric currents effectively communicate magnetic stresses along the planetary magnetic-field lines, and isorotation is more likely to hold. In this case, the field remains poloidal, and the plasma remains co-rotating. The same effect occurs in pulsar magnetospheres, although here relativistic effects impact the communication between the footpoints of the field on the neutron star and the magnetosphere (Goldreich and Julian 1969; Uzdensky 2003).

Ferraro’s isorotation theorem and these examples illustrate how magnetic fields resit tangling in plasma systems through tension forces and the exchange of magnetic stresses. In the case of the solar wind, the interplanetary magnetic field is not poloidal and winds up by advection (Parker 1958b). It develops toroidal components and magnetic tension that exchange angular momentum between the field and the flow, ultimately slowing down the rotation of the Sun (Weber and Davis 1967; Verscharen et al. 2021).

## Modulation of Parallel Electron Heat Flux by Kinetic Instabilities

In plasmas with low levels of collisions, electrons often carry significant heat flux due to their high mobility (Hollweg 1974; Feldman et al. 1976). The electron heat-flux vector is defined as the third moment of the electron velocity distribution function $f_{\mathrm {e}}$:

14 $$ \boldsymbol{q}_{\mathrm {e}}=\frac{m_{\mathrm {e}}}{2}\int \left ( \boldsymbol{v}-\boldsymbol{U}_{\mathrm {e}}\right )\left ( \boldsymbol{v}-\boldsymbol{U}_{\mathrm {e}}\right )^{2}f_{\mathrm {e}}\, \mathrm {d}^{3}v, $$

where $\boldsymbol{U}_{\mathrm {e}}$ is the electron bulk velocity, i.e., the first velocity moment of $f_{\mathrm {e}}$. Electron heat transfer is dominated by heat flow along magnetic-field lines. When plasma processes create a non-zero divergence of $\boldsymbol{q}_{\mathrm {e}}$, the heat flux contributes to a local change in thermal energy density (Coburn et al. 2026). Therefore, the behaviour of heat flux is of great interest to our understanding of plasma energetics.

In the solar wind, the electron heat flux can be large (Scime et al. 1999; Cranmer and Schiff 2021; Halekas et al. 2021) and drive plasma instabilities (Gary et al. 1975, 1999; Verscharen et al. 2022). These collisionless processes compete with the collisional regulation of the heat flux according to the Spitzer–Härm theory (Spitzer and Härm 1953) in the solar wind (Bale et al. 2013). In a wide range of astrophysical plasmas, heat conduction plays an important role, for instance, during galaxy formation and in the intracluster medium (e.g., Fabian et al. 2002). In the case of the intracluster medium, thermal conduction from the hot outskirts into the radiatively cooling core may help stave off a cooling catastrophe (Zakamska and Narayan 2003; Kim and Narayan 2003), which would otherwise lead to catastrophic mass accretion rates of up to a thousand solar masses per year (Fabian and Nulsen 1977). In fact, X-ray spectroscopic observations of galaxy clusters (Fabian et al. 2002) indicate typical cold-mass accretion rates onto the central dominant galaxy of tens, and at most hundreds, of solar masses per year (e.g., Peterson et al. 2003), a puzzle known as the *cooling-flow problem* (Peterson and Fabian 2006).

Complicating the thermal regulation of the cooling intracluster medium are indications that heat conduction in such environments can be suppressed by factors of $\sim 0.1$ to $\sim 10^{-2}$ relative to the Spitzer–Härm prediction (Binney and Cowie 1981; Ettori and Fabian 2000; Markevitch et al. 2003). Similar levels of heat-flux suppression are also observed in laboratory experiments (Meinecke et al. 2022) and first-principles numerical simulations (see Coburn et al. 2026 for a review). All these transport effects occur in tangled and turbulent magnetic-field configurations, which further complicate any reductions in the potency of the conductive heat flux. Understanding whether and to what extent electromagnetic fields are ordered or tangled in different space and astrophysical contexts therefore has deep implications for heat transport and for the acceleration of, for instance, cosmic-ray electrons (and ions) in shock waves (e.g., Morlino et al. 2021) and by second-order (stochastic) Fermi processes (Brunetti and Vazza 2020) in various heliospheric and astrophysical environments.

All instabilities discussed in Sect. 2.2.1 as well as an additional family of electron-resonant instabilities (Verscharen et al. 2022) have the potential to modify electron transport parallel to the magnetic field and thus also the electron heat flux. A detailed discussion of electron-mediated heat transport in space, astrophysical, and laboratory plasmas by collisions and collisionless processes including kinetic instabilities is provided in the companion review to this article by Coburn et al. (2026). Electron-driven instabilities and their impact on the evolution of the electron distribution function are discussed in the companion review to this article by Jiang et al. (2026).

## Electron Trapping in Inhomogeneous Magnetic Fields

We now describe the dynamics of electrons that are trapped in a local minimum of the magnetic-field strength while the gyro-centres of their trajectories move parallel or anti-parallel to the field. An electron passing adiabatically through such an inhomogeneous magnetic field changes its pitch-angle $\theta $, where $\cos \theta = v_{\parallel}/v$. While the electron conserves its kinetic energy $W$ and magnetic moment $\mu =W\sin ^{2}\theta /B$, the modulation of $\theta $ is determined by the spatially varying magnetic-field strength $B$. In a local depletion of $B$, a magnetic bottle can form, which traps part of the electron distribution.

The modulation of electron trajectories due to a changing magnetic field causes changes in the electron velocity distribution. The evolving velocity distribution can, especially before achieving a steady state, contain sufficient free energy to excite microinstabilities. In this case, trapping is an example of a process through which structures with large scales (ion scales or larger) couple with electron-scale structures through the kinetics of the electron distribution. Similar physical processes occur over a range of scales and in different plasma environments. In this section, we show examples from the Earth’s magnetosphere and magnetosheath as these environments provide directly observable space plasmas in which electron trapping plays an important role.

### Trapping in the Inner Magnetosphere

The Earth’s inner magnetosphere and the magnetospheres of planets with strong magnetic fields in general are natural large-scale magnetic traps due to the inhomogeneity of their magnetic fields. Electrons gyrate around the approximately dipolar field lines of the magnetosphere. The magnetic field strength $B$ is at a minimum in the equatorial plane and at maximum at the foot points of the field lines near the planet’s poles.

In a strong magnetic field, the electron gyro-radius $\rho _{\mathrm {e}}$ is sufficiently small to neglect the gyro-motion in the description of the particle dynamics. Effects due to the finite $\rho _{\mathrm {e}}$ in the context of magnetic trapping are discussed in Sect. 4.4.3. Under the small-$\rho _{\mathrm {e}}$ assumption, we discuss two types of motion: particle oscillations along magnetic-field lines and particle cross-field drift due to curvature and gradient effects (Northrop 1963). Due to the azimuthal symmetry of magnetic dipole field, cross-field drift results in a quasi-periodic particle motion around the planet (for a discussion of exceptions, see Sect. 4.3). We thus focus on the bounce motion in the context of particle trapping and de-trapping.

**Fig. 8:**
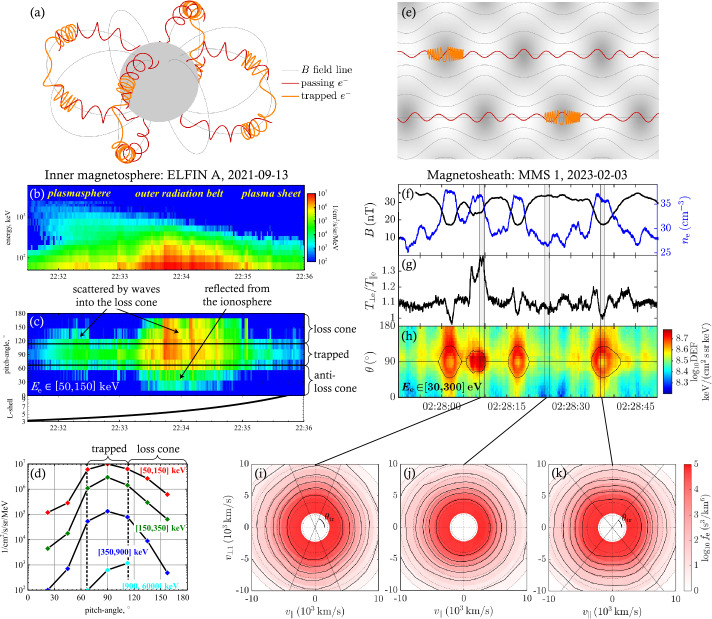
Trapping in inhomogeneous magnetic-field structures. (a) Passing/escaping (red) and trapped (orange) electrons in a planetary dipole magnetic field. (b) Spectra of trapped electrons measured by the ELFIN CubeSat (Angelopoulos et al. 2020) at different locations (for details, see Artemyev et al. 2022). (c) Pitch-angle distribution of $[50,150]$ keV electrons for the same interval as in (b) with indication of trapped, scattered, and reflected populations (for details, see Mourenas et al. 2021). (d) Pitch-angle distribution measured by ELFIN around 22:33:41 UT. (e) Trapping in ion-scale magnetic structures. The gray shading indicates $B$, the gray curves represent magnetic-field lines. The electron distribution is separated into passing/escaping (red) and trapped (orange) electrons. (f) MMS measurements of an ion-scale structure in the magnetosheath with anti-correlation in $B$ and $n_{\mathrm {e}}$, typical for mirror modes. (g) Electron temperature anisotropy $T_{\perp \mathrm {e}}/T_{\parallel \mathrm {e}}$ for the same interval as in (f). (h) Differential energy flux of $[30,300]$ eV electrons for the same interval as in (f). (i)–(k) Electron velocity distribution functions $f_{\mathrm {e}}(v_{\parallel},v_{\perp 1})$ at the three times indicated in (h)

Figure 8a shows examples of trapped and untrapped particle orbits in the inner magnetosphere. In the absence of spatial and temporal perturbations on scales comparable to the gyro-radius, the magnetic moment $\mu $ is conserved (Landau and Lifshitz 1988; Lichtenberg and Lieberman 1983). The particle bounce motion is then a one-dimensional oscillation in an effective potential well $\mu B(s)$, where $B(s)$ is the magnetic-field magnitude as a function of the field-aligned spatial coordinate $s$. We define $s=0$ as the location at which the magnetic field assumes its minimum value; i.e., at the magnetic equator.

This configuration allows us to use an analogy with the classical nonlinear pendulum (Lichtenberg and Lieberman 1983). We introduce two regions in phase space, depending on the energy $W$ and pitch-angle $\theta $ of the particles. We define a maximum $B$-value $B_{\max}$ in such a way that particles with energy $W$ greater than $\mu B_{ \max}$ escape from the oscillatory motion. These escaping particles are illustrated by the red trajectories denoted as *passing* in Fig. 8a. Particles with energy $W<\mu B_{\max}$ remain in their oscillatory motion and are thus trapped between the two locations $s_{\pm}$, which are defined by $W=\mu B(s_{\pm})$. The trapped particles are illustrated by the orange trajectories in Fig. 8a. For planetary magnetospheres, $B_{\max}$ is generally given by the magnetic field magnitude at those altitudes at which particle collisions with the dense ionosphere and neutrals result in particle losses (e.g., about $\sim 100$ km in the Earth’s magnetosphere).

Taking into account that, in a dipole field, $B$ reaches its minimum value at the equator, the passing/escaping population consists of particles with small equatorial pitch-angles $\theta < \theta _{\mathrm{tr}}$ and $\theta > (180^{\circ }- \theta _{\mathrm{tr}})$, where

15 $$ \sin \theta _{\mathrm{tr}}(s)=\sqrt{\frac{B(s)}{B_{\max}}} $$

defines the trapping angle $\theta _{\mathrm{tr}}(s)$. The trapped electrons are those with larger pitch-angles ($\theta _{\mathrm{tr}} < \theta < 180^{ \circ }- \theta _{\mathrm{tr}}$) that are reflected when $B$ increases towards its maximum value $B_{\max}$.

As the passing electrons are ultimately lost to the collisional ionosphere and atmosphere, the result of this process is an electron distribution that consists only of the trapped population at all $|s|<|s_{\pm}|$. The empty part of phase space with smaller pitch-angles defines the *loss cone*. The pitch-angle $\theta _{\mathrm{tr}}$ of the boundary of the loss cone at a given $s$ depends on the magnetic-field strength $B_{\max}$ at the mirror point $s_{\pm}$ and the magnetic-field strength $B(s)$ at position $s$ according to Eq. (15); i.e., the angle depends on latitude and drift shell in a planetary field (e.g., Koskinen and Kilpua 2022).

**Fig. 9:**
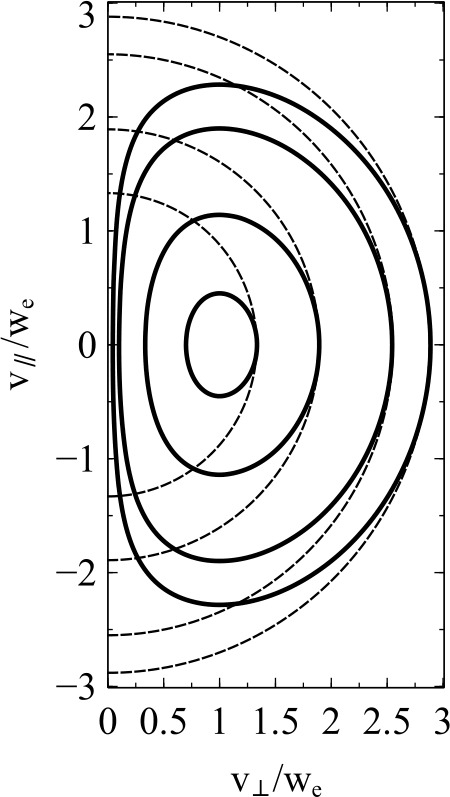
Illustration of the loss-cone distribution in Eq. (16) with $a=1$ (solid curves). For comparison, we overplot the corresponding Maxwellian distribution without a loss cone ($a\rightarrow0$, dashed curves). The distributions are visualised as contours of constant phase space density

A simple analytical form of the loss-cone distribution is given by

16 $$ f_{\mathrm {e}}\left ( v_{\perp},v_{\parallel}\right ) = \frac{{n_{0} v_{\perp }^{2a} }}{{\pi ^{3/2} a\Gamma (a)w_{\mathrm {e}}^{3 + 2a} }} \exp \left ( { - \frac{{v_{\perp}^{2}+v_{\parallel }^{2} }}{{w_{\mathrm {e}}^{2} }}} \right ), $$

where $\Gamma (x)$ is the $\Gamma $-function and $a>0$ is a constant parameter that determines the size of the loss cone (see Fig. 9).

The *bounce period* is given by (Lyons and Williams 1984)

17 $$ \tau _{\mathrm {b}}=\sqrt{2m_{\mathrm {e}}}\int \limits _{s_{-}}^{s_{+}} \frac{1}{\sqrt{W-\mu B(s)}}\,\mathrm {d}s=\sqrt{ \frac{2m_{\mathrm {e}}}{W}}\int \limits _{s_{-}}^{s_{+}} \frac{1}{\sqrt{1-\displaystyle \frac{B(s)}{B(0)}\sin ^{2}\theta }}\, \mathrm {d}s, $$

where the second equality follows from $\mu =W\sin ^{2}\theta /B(0)$. For relativistic particles, $2m_{\mathrm {e}}/W\to 1/c^{2}$ in Eq. (17), and then the bounce period depends on pitch-angle only but not on energy. We find that $\tau _{\mathrm {b}}\sim \ell /\sqrt{2W/m_{\mathrm {e}}}$ where $\ell \approx (s_{+}-s_{-})$ is the system’s spatial scale along magnetic-field lines. Figure 8 shows measurements of trapped and escaping populations of electrons at different distances from the equator in the Earth’s magnetosphere.

### Trapping in Ion-Scale and Electron-Scale Magnetic-Field Structures

The concept of a loss cone can be generalised to any magnetic-field configuration with a local minimum in $B$. Thus, loss-cone-like distributions arise also in other local magnetic-field structures, such as mirror modes or magnetic holes. Figure 8e illustrates the trapping and passing of electrons in a quasi-period ion-scale structure (e.g., a chain of mirror modes). The magnetic-field strength $B$ follows a spatial modulation, represented by the varying levels of gray shading. Due to the scale separation between the size of the ion-scale structure and $\rho _{\mathrm {e}}$, the electrons follow adiabatic trajectories in this field configuration. Electrons with $\theta _{\mathrm{tr}}<\theta <(180^{\circ}-\theta _{\mathrm{tr}})$ are trapped in the ion-scale structures, while electrons with $\theta < \theta _{\mathrm{tr}}$ or $\theta >(180^{\circ}- \theta _{\mathrm{tr}})$ pass through the ion-scale structures.

Figure 8f shows an example of mirror modes measured by the MMS spacecraft in Earth’s magnetosheath behind a quasi-perpendicular bow shock. Mirror modes are non-propagating pressure-balanced structures consisting of compressive magnetic-field fluctuations (peaks and troughs in the field magnitude) accompanied by anti-correlated fluctuations in thermal pressure (seen as density troughs and peaks). The characteristic size of mirror modes is of order the ion gyro-radius and typically observed on timescales of 3-24 s in the magnetosheath (Soucek et al. 2008). In the associated magnetic-field troughs, part of the electron population are trapped and form a loss-cone-like distribution.

Figure 8h shows the modulation of the electron pitch-angle distribution in the mirror-mode structures shown in Fig. 8f. The black curves represent the trapping angle as $\theta _{\mathrm{tr}}$ and $(180^{\circ}-\theta _{\mathrm{tr}})$ from Eq. (15). The part of the distribution between these curves corresponds to trapped particles, while the part outside these curves corresponds to the loss cone of passing particles. We take $B_{\max}$ to be the average $B$ across the interval (see Yao et al. 2018). Below panel (h), we show two-dimensional cuts in $(v_{\parallel},v_{\perp 1})$ space of the electron velocity distribution $f_{\mathrm {e}}$ at three times during the interval, where $v_{\perp 1}$ is one of the Cartesian velocity axes in the plane perpendicular to $\boldsymbol{B}$. The straight lines in these figures indicate the separation between trapped (at small $|v_{\parallel}|$) and passing (at large $|v_{\parallel}|$) particles. The first distribution (Fig. 8i) shows that the trapped population in the magnetic minimum of this mirror mode exhibits larger values of $f_{\mathrm {e}}$ at large pitch-angles ($\theta _{\mathrm {t}r}<\theta <180^{ \circ}-\theta _{\mathrm{tr}}$). This behaviour translates to a high electron temperature anisotropy with $T_{\perp \mathrm {e}}/T_{\parallel \mathrm {e}}>1$ (see also Fig. 8g).

The second distribution (Fig. 8j) is taken outside the mirror mode. It is more isotropic than inside the field depletion as shown by the solid black isocontours that lie close to the dotted black contours of constant energy. Consequently, this distribution has a lower $T_{\perp \mathrm {e}}/T_{\parallel \mathrm {e}}$ than the distribution inside the structure.

**Fig. 10:**
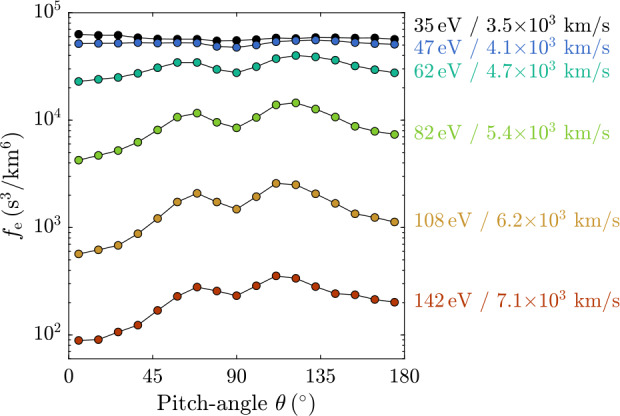
Butterfly distribution measured by MMS in the Earth’s magnetosheath, same distribution as in Fig. 8. Each curve shows $f_{\mathrm {e}}$ as a function of pitch-angle $\theta $ for different electron energies/speeds as indicated by colours

Magnetic-field minima can also reshape the electron distribution in other ways. An example is shown by the third distribution (Fig. 8k). Here, $f_{\mathrm {e}}$ is reduced at small pitch-angles, $\theta < \theta _{\mathrm {t}r}$ and $\theta >(180^{\circ}- \theta _{\mathrm {t}r})$, like in the loss cone shown in the first distribution. The contours of $f_{\mathrm {e}}$ also exhibit a constriction at $\theta \approx 90^{\circ}$. This distribution corresponds to the so-called *butterfly* or *doughnut distribution*, which is often found in mirror-mode structures in the magnetosheath downstream the quasi-perpendicular bow shock (Yao et al. 2018) and in local magnetic-field minima downstream the quasi-parallel bow shock (e.g., Svenningsson et al. 2022). Figure 10 shows an example of such a butterfly distribution for different energies. A possible explanation for this shape involves the combination of betatron and Fermi acceleration and deceleration processes as the mirror mode grows deeper (Yao et al. 2018; Jiang et al. 2022). Despite the presence of the loss cone and other strong non-equilibrium features in the distribution in Fig. 8k, $T_{\perp \mathrm {e}}/T_{\parallel \mathrm {e}} \approx 1$.

It is important to consider the exact shape of the distribution when studying secondary instabilities, as two distributions with the same $T_{\perp \mathrm {e}}/T_{\parallel \mathrm {e}}$ can indeed have very different phase-space features and thus kinetic properties including their potential to drive microinstabilities. For instance, the trapping of electrons can excite whistler waves within mirror-mode structures, which in turn re-shape the electron distribution (Kitamura et al. 2020; Jiang et al. 2022; Svenningsson et al. 2022; Jiang et al. 2024). In the solar wind, when a core–strahl electron configuration encounters ion-scale magnetic holes, the suprathermal part of the electron distribution can be modulated in such a way that it emits Langmuir waves through the *bump-on-tail instability* (Boldú et al. 2023; Liu et al. 2025). Through the scattering with the growing Langmuir waves, some electrons then transition from the passing strahl population into the trapped population. These examples show that self-regulated wave–particle interactions through secondary instabilities can play a critical role in the multi-scale dynamics of trapped electrons in nonlinear structures.

Butterfly distributions are also regularly observed in Earth’s radiation belts; however, it is likely that other processes are responsible for the minima at pitch-angles near $90^{\circ}$ in these regions. At large $L$-shells, they are probably caused by drift-shell splitting and magnetopause shadowing (Ni et al. 2020). At low $L$-shells, they are caused by local acceleration due to wave–particle interactions (Xiao et al. 2015).

Electron trapping can also occur in coherent electron-scale structures that are frequently observed in space plasmas (Huang et al. 2017; Yao et al. 2017; Liu et al. 2019; Zhou et al. 2019) and sometimes occur nested inside ion-scale structures (Li et al. 2020). For instance, observations by MMS in the Earth’s magnetosheath reveal flux enhancements in the electron pitch-angle distribution near the local maximum of the magnetic-field strength (Xie et al. 2024). Trapping by the magnetic mirror force cannot explain this behaviour. Instead, an inhomogeneity of the electric potential along the direction of the magnetic field associated with these structures may potentially explain this electrostatic trapping effect at electron-scale structures. In this scenario, the electric potential in combination with the mirror force transforms the initially isotropic electron distribution into a trapped and a streaming population.

### Trapping by Transient Magnetic-Field Enhancements

A global dipole magnetic field causes charged particles to follow a closed azimuthal drift motion around the planet along contours of constant equatorial field (see Roederer 1970). In open systems, however, gradient drifts can result in particle losses through the system boundaries. The Earth’s bow shock and the Earth’s magnetotail are two examples of plasma systems with such boundaries. Here, the spatial limitations of the systems along the drift direction can result in particle escape.

An effective mechanism of particle trapping in such systems occurs in the form of local magnetic-field peaks, changing the drift trajectories and thus trapping particles in analogy to closed drift trajectories in dipole fields. Simulations reveal this kind of trapping of ions and electrons in the Earth’s magnetotail (Gabrielse et al. 2017; Ukhorskiy et al. 2018). Nonlinear dipolarisation fronts associated with transient magnetic reconnection form the magnetic-field enhancements in this case (see Nakamura et al. 2004; Runov et al. 2009; Sitnov et al. 2009).

Similar trapping mechanisms may also manifest in planetary foreshocks when spatially localised transients form magnetic-field enhancements (e.g., Omidi et al. 2010; Turner et al. 2013). The main feature of this trapping process is the transport of the trapped particles along with the magnetic-field enhancements. The particles can be energised through adiabatic betatron heating if the transport carries them across increases in the background magnetic field (Gabrielse et al. 2017; Ukhorskiy et al. 2018).

Wave–particle interactions can lead to the de-trapping of particles from such magnetic-field configurations. When undergoing these resonant interactions, particles do not conserve their magnetic moment any more, leading to a change in their drift orbit or to scattering due to magnetic-field curvature (see Sect. 4.4.3).

### De-Trapping of Electrons

Particles that are trapped in steady-state field structures remain trapped as long as their motion is adiabatic. However, wave–particle interactions, microinstabilities, the betatron effect, scattering by magnetic-field rotations, and collisions can transfer particles from the trapped population into the escaping loss cone of the distribution. These processes lead to the *de-trapping* of particles.

#### De-Trapping Through Wave–Particle Interactions

Particles can transition from the trapped to the escaping region of velocity space through particle scattering with fluctuating electromagnetic fields. In order for these fields to disrupt the conservation of the magnetic moment $\mu $, they often have spatial and/or temporal scales comparable to the scales of the gyro-motion of the particles. Resonant wave–particle interactions are a sub-family of such processes (Schulz and Lanzerotti 1974; Lyons and Williams 1984).

Electrons resonate with a wave when they fulfil the resonance condition (Lichtenberg and Lieberman 1983; Lyons and Williams 1984)

18 $$ \omega _{\mathrm {r}}=k_{\parallel}v_{\parallel}+n\Omega _{\mathrm {e}} $$

introduced more generally in Eq. (13). For $n=0$, the resonance condition describes the *Landau resonance*; for all other $n$, it describes *cyclotron resonances* of different orders. Equation (18) suggests that waves with frequency $\omega _{\mathrm {r}} \sim n|\Omega _{\mathrm {e}}|$ are very effective in scattering particles, viz., by covering a large range in resonant $v_{\parallel}$. Indeed, in the Earth’s inner magnetosphere, electrons are efficiently scattered by electron-harmonic waves with $\omega _{\mathrm {r}}\sim n|\Omega _{\mathrm {e}}|$ and $n=1,2,3,\dots $ (e.g., Zhang et al. 2015; Ni et al. 2016), whistler waves with $\omega _{\mathrm {r}}\lesssim |\Omega _{\mathrm {e}}| $ (e.g., Mourenas et al. 2012; Ni et al. 2016), fast-magnetosonic/whistler waves with $\omega _{\mathrm {r}} \ll |\Omega _{\mathrm {e}}|$ and $\omega _{\mathrm {r}}\sim k_{\parallel }v_{\parallel}$ (Horne et al. 2007; Mourenas et al. 2013), and electrostatic broad-band waves with $\omega _{\mathrm {r}} \sim n|\Omega _{\mathrm {e}}| $ and $n=1,2,3,\dots $ (Vasko et al. 2018; Shen et al. 2021).

**Fig. 11:**
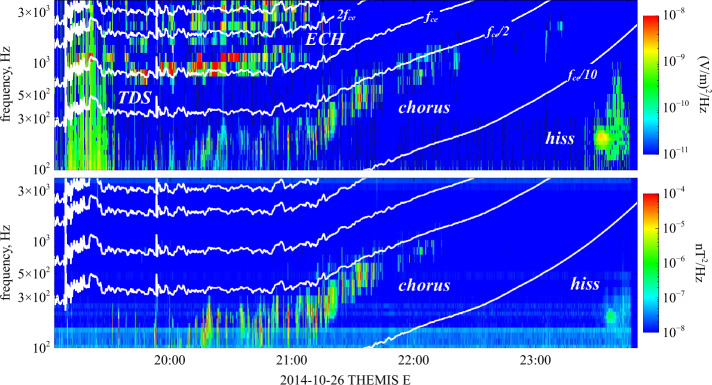
Spectrograms of the main wave modes responsible for electron scattering into the loss cone in the inner magnetosphere measured by THEMIS E (Angelopoulos 2008). The top panel shows the electric-field spectrum, and the bottom panel shows the magnetic-field spectrum (Bonnell et al. 2008; Le Contel et al. 2008). The white curves show electron-cyclotron harmonics $nf_{\mathrm{ce}}$ and fractions $f_{\mathrm{ce}}/n$, where $f_{\mathrm {ce}}= |\Omega _{\mathrm {e}}|/2\pi $, measured by the fluxgate magnetometer (Auster et al. 2008). The main wave modes are identified, see text for definitions

Figure 11 shows a measured spectrogram of wave modes that affect the electron dynamics and result in the scattering of electrons into the loss cone.^2^ Time-domain structures (TDS; Mozer et al. 2015) are electrostatic nonlinear solitary waves observed as broad-band noise that effectively scatters electrons with $W<10$ keV (Shen et al. 2024). Part of this broad-band electrostatic noise can be due to short-wavelength kinetic Alfvén waves (Chaston et al. 2012) that effectively scatter electrons with $W\sim 100$ keV (Shen et al. 2023). Electron-cyclotron harmonics (ECH; Zhang et al. 2015) are electrostatic high-frequency waves that effectively scatter electrons with $W<1$ keV (Ni et al. 2016). Whistler chorus and hiss waves (Agapitov et al. 2013) effectively scatter electrons with energies between 10 and 1000 keV (Artemyev et al. 2013). Similar resonant wave–particle interactions are responsible for ion scattering in the Earth’s inner magnetosphere. Ion-cyclotron waves with $\omega _{\mathrm {r}}\leq \Omega _{\mathrm {i}}$ and fast-magnetosonic/whistler waves with $\omega _{\mathrm {r}}\sim n\Omega _{\mathrm {i}}$, where $\Omega _{\mathrm {i}}$ is the cyclotron frequency of the ion species under consideration, effectively scatter ions (Ma et al. 2019) and transfer them into the loss cone. Pitch-angle scattering and resulting de-trapping of electrons by whistler waves can also occur in the transition layer of Earth’s bow shock (Katou and Amano 2019; Amano et al. 2020; Lindberg et al. 2024).

The diffusion coefficient

19 $$ D_{\theta \theta} (\theta ) = \frac{\langle (\Delta \theta )^{2}\rangle}{\tau} $$

measures the efficiency of resonant particle scattering in pitch-angle by plasma waves (Andronov and Trakhtengerts 1964; Kennel and Engelmann 1966). When evaluated at the trapping angle, $D_{\theta \theta} (\theta _{\mathrm{tr}})$ quantifies the corresponding efficiency of particle losses from the magnetic trap. In this definition, $\Delta \theta $ is the pitch-angle change for a single resonant interaction, and $\tau $ is the average time between two successive resonant interactions. $D_{\theta \theta}$ is measured in degrees^2^/second or radians^2^/second. The timescale of particle de-trapping, i.e., the time it takes diffusion to transport particles into the loss cone, $\theta \to \theta _{\mathrm{tr}}$, is approximately $\tau _{\mathrm {L}}\sim 1/ D_{\theta \theta} (\theta _{\mathrm{tr}})$ when $D_{\theta \theta}$ is measured in radians^2^/second (Albert and Shprits 2009).

*Quasilinear theory* is a framework that allows us to derive diffusion coefficients under the assumption that each $\Delta \theta $ is much smaller than $\theta $ and that the particle orbits are small perturbations to the unperturbed particle trajectories in the background field $\boldsymbol{B}_{0}$ (Drummond and Pines 1962; Vedenov et al. 1962). The resulting diffusion coefficient is linearly proportional to the wave intensity, $D_{\theta \theta} \propto B_{\mathrm {w}}^{2}$, where $B_{\mathrm {w}}$ is the magnetic-field amplitude of the resonant wave (Schulz and Lanzerotti 1974; Lyons and Williams 1984). The applicability of the quasilinear approach is largely constrained to small wave intensities, $B_{\mathrm {w}}^{2}/B_{0}^{2}\ll 1$, so that the magnetic-field perturbations (and the electric-field perturbations) remain much smaller than the background magnetic field $B_{0}$. For a sufficiently long time $\sim \tau _{\mathrm {L}}$, even these small perturbations transfer a significant number of particles from the magnetic trap into the loss cone. The approximation of unperturbed trajectories is violated in the case of very intense waves (for thresholds of wave amplitudes, see Karpman 1974; Zhang et al. 2022), when nonlinear resonant effects become important (Albert et al. 2013; Artemyev et al. 2024a).

**Fig. 12:**
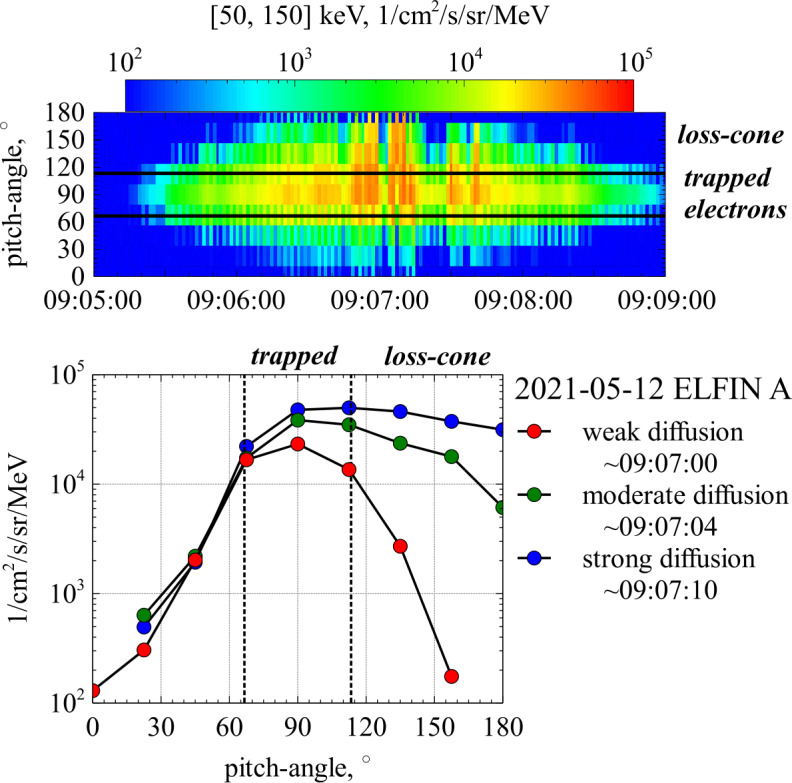
Typical electron pitch-angle distributions in the inner magnetosphere measured by ELFIN (Angelopoulos et al. 2023). The top panel shows pitch-angle distributions around afternoon magnetic local time. Bursts of electron fluxes within the loss cone are due to electron scattering by whistler waves (Tsai et al. 2022). The bottom panel shows three pitch-angle distributions with different filling rates of the loss cone. The distribution with the filled loss cone is likely formed by electron scattering in the strong diffusion limit

The strongest rate of particle losses from magnetic traps occurs in the *strong diffusion regime*, i.e., when $\tau _{\mathrm {L}}$ is comparable to the bounce period $\tau _{\mathrm {b}}$. This regime is characterized by $D_{\theta \theta}\approx \theta _{\mathrm{tr}}^{2}/\tau _{\mathrm{b}}$ (Kennel 1969) and $\theta _{\mathrm{tr}}={\mathrm{arcsin}}\left (B(0)/B_{\max}\right ) \approx B(0)/B_{\max}\ll 1$. In this regime, the loss cone with $\theta <\theta _{\mathrm{tr}}$ and $\theta >(180^{\circ}-\theta _{\mathrm{tr}})$ is continuously filled with particles through a flow of particles from the magnetic trap. It is the strongest loss regime that can be described by diffusion, and it is achieved when $B_{\mathrm {w}}$ is sufficiently large. A further increase of the wave intensity would not change the flow of particles from the trap. Figure 12 shows electron pitch-angle distributions in regimes of weak and strong diffusion for electrons scattered by whistler waves in the Earth’s inner magnetosphere. For very large wave intensity, the quasilinear diffusion approximation breaks down, and other nonlinear resonant effects overfill the loss cone through a flow of particles from the trap that exceeds the strong diffusion limit (Zhang et al. 2022).

#### Self-Limitation of Trapping

An important concept related to magnetic traps and particle escape from these traps is the *self-limitation of trapping*. The magnetic-trap configuration assumes the presence of a loss cone that is empty or depleted in the absence of strong particle scattering. Therefore, the particle velocity distribution is not isotropic and contains a flux gap within a small pitch-angle range. This non-equilibrium feature leads to a loss-cone anisotropy, which is a source of free energy for instabilities (Sagdeev and Shafranov 1961). Trapped electrons then self-consistently generate the waves responsible for their de-trapping by scattering as described in Sect. 4.4.1. This self-induced scattering into the loss cone occurs, for example, when the temperature anisotropy is large (Kennel and Petschek 1966) or when butterfly distributions are present (Kitamura et al. 2020; Svenningsson et al. 2022). The bottom panels of Fig. 8 show example cases of non-equilibrium distributions in a trapping field configuration. Since these types of distributions can result from trapping, whistler waves generated by instabilities are regularly found in mirror-mode structures (Smith and Tsurutani 1976). Estimates of pitch-angle diffusion coefficients reveal that whistler waves significantly impact the electron velocity distributions throughout the Earth’s magnetosheath (Svenningsson et al. 2024).

Under many plasma conditions, the loss-cone distribution in Eq. (9) is unstable to wave generation. The free energy for these instabilities is comparable to the free energy due to the associated temperature anisotropy (Shklyar and Matsumoto 2009). Magnetic-trap configurations with such a loss-cone electron distribution are often unstable to whistler-wave and electron-cyclotron harmonic wave instabilities (Kennel and Petschek 1966; Karpman et al. 1975; Ashour-Abdalla and Kennel 1978; Liu et al. 2018), which self-consistently scatter particles into the loss cone. For a fixed loss-cone size determined by the magnetic-field configuration, the wave growth rate is proportional to the density of the trapped particles, and thus stronger waves are generated when there are more particles inside the trap. This self-regulating mechanism prevents the flux of trapped particles to increase unboundedly (Kennel and Petschek 1966; Summers et al. 2009; Mourenas et al. 2024).

The betatron effect provides an additional self-limiting process for trapped particle fluxes in magnetic traps. Any adiabatic variation of the magnetic configuration of the trap, i.e., a slow change of the $B(s)$ profile in time, results in adiabatic variations of the charged-particle energy $W$. While conserving the magnetic moment $\mu $, the betatron effect modifies the energy of the trapped electrons, mediated by an electric field that must obey Faraday’s law,

20 $$ \boldsymbol{\nabla }\times \boldsymbol{E}=-\frac{1}{c} \frac{\partial \boldsymbol{B}}{\partial t}, $$

associated with the slow time-dependence of $\boldsymbol{B}$. This electric field does work on the trapped particles and modifies their energy $W$. The conservation of the second adiabatic invariant $\propto \oint{\sqrt{W-\mu B}\,\mathrm {d}s}$, which is associated with the bounce motion (Schulz and Lanzerotti 1974), results in a variation of the parallel particle energy, analogously to Fermi acceleration (Bogachev and Somov 2005; Artemyev et al. 2012; Borissov et al. 2016; Lichko and Egedal 2020). Such an adiabatic energy change results in the formation of anisotropic distribution functions that can become unstable to wave generation (Jiang et al. 2022; Frantsuzov et al. 2024). The subsequent particle scattering by the unstable waves also leads to losses from the magnetic trap as described in Sect. 4.4.1.

In most situations, particle distributions in trapping field configurations have higher particle fluxes outside the loss cone, so that wave–particle interactions mostly result in particle scattering towards and into the loss cone. However, resonant particle scattering can also work in the opposite direction, transferring particles from the loss cone into the trapped regime. In this case, the wave–particle interactions support and enhance the trapped population (Liu et al. 2025) and even allow particles to spend more time trapped in systems with large loss cones. This process is important for particle acceleration at shock waves. Particles crossing the shock or reflecting at the shock are lost and do not return for further acceleration. However, if these particles are scattered by waves after their interaction with the shock, they may return, providing them with an opportunity for further acceleration (Amano et al. 2022).

#### Electron Scattering by Magnetic-Field Rotations

**Fig. 13:**
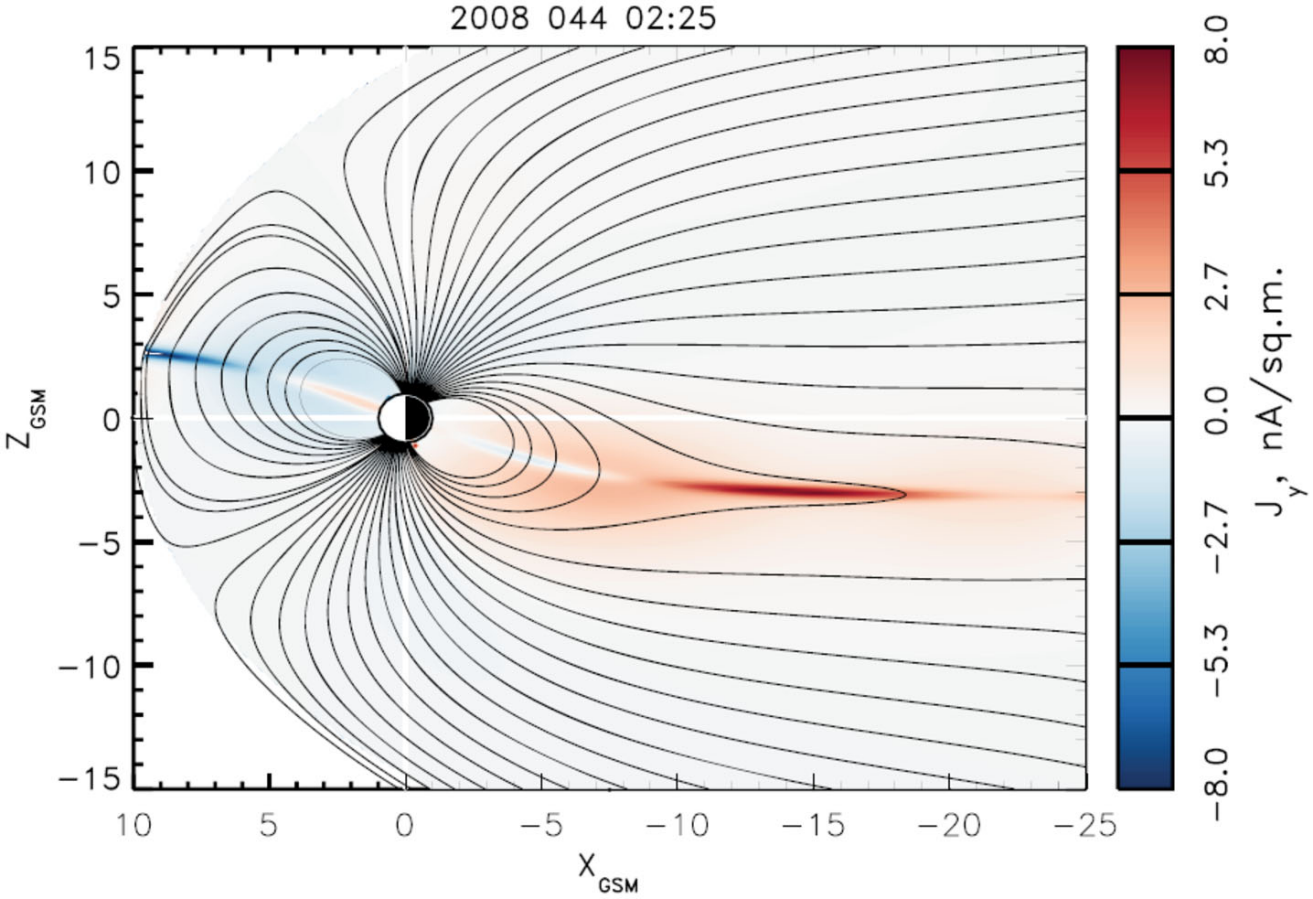
Magnetic-field configuration and current density in Earth’s magnetosphere. The magnetic-field lines are shown in black and the out-of-plane current density in colour. The most intense current density occurs in the equatorial magnetotail, where magnetic-field lines have small curvature radii. Particle scattering at small magnetic-field curvature radii de-trap particles in the magnetosphere. Adopted from Sitnov et al. (2019) and with permission by John Wiley and Sons

Besides the scattering of particles by electromagnetic waves, magnetic-field curvature also has the potential to scatter particles (Chirikov 1987). This scattering is known as *current-sheet scattering* since large rotations in the magnetic field are associated with strong currents according to Ampère’s law. In the magnetosphere, a large-scale current sheet where this effect is prevalent is associated with the magnetic-field curvature in the equatorial magnetotail, see Fig. 13.

The efficiency of the current-sheet scattering mechanism for electrons is controlled by the ratio of the curvature radius $R_{\mathrm {c}}$ of the magnetic-field lines to the electron gyro-radius $\rho _{\mathrm {e}}$. If $R_{\mathrm {c}}/\rho _{\mathrm {e}}\gg 1$, the scattering is exponentially weak with $D_{\theta \theta} \propto \exp (-R_{\mathrm {c}}/\rho _{\mathrm {e}}) \ll 1$ (Birmingham 1984; Neishtadt 2000). If $R_{\mathrm {c}}/\rho _{\mathrm {e}} \ll 1$, the scattering is weak and depends on $R_{\mathrm {c}}/\rho _{\mathrm {e}}$ as $D_{\theta \theta} \propto R_{\mathrm {c}}/\rho _{\mathrm {e}} \ll 1$ (Büchner and Zelenyi 1989). If $R_{\mathrm {c}}/\rho _{\mathrm {e}}\sim 1$, the scattering is most effective, providing an almost stochastic motion of the particles (Horton 1997; Zelenyi et al. 2013). When acting, this scattering mechanism traps or de-traps particles. Several examples of current-sheet scattering are found in planetary magnetotails (e.g., Delcourt et al. 1994; Sergeev et al. 2012; Artemyev et al. 2024b) and in the solar wind (e.g., Artemyev et al. 2020; Malara et al. 2021).

#### Electron Scattering by Collisions

If the rate of particle collisions between the trapped particles and other particles (ions, electrons, and/or neutral particles) is sufficiently high, collisions are capable of de-trapping particles by scattering them into the loss cone. In this case, elastic and inelastic scattering changes the pitch-angle and energy of the trapped particles, eventually resulting in particle escape from the trap. Energetic electrons in the Earth’s inner radiation belt, for example, are trapped very close to the planet: at less than 3 Earth radii in the equatorial plane. These electrons encounter dense ionospheric plasma consisting of heavy ions and free electrons during their bounce motion (see Selesnick 2012, 2016, and references therein). The resulting collisions between the energetic electrons and the ionospheric plasma lead to the de-trapping of some of the energetic radiation-belt electrons.

Elastic electron collisions with ions do not change the energies $W$ of the participating electrons but scatter their pitch-angles $\theta $. The differential cross-section is the key parameter controlling the efficiency of this pitch-angle scattering. It describes the probability of a scattering event at a specific solid angle and depends on the electron energy $W$, scattering angle $\Delta \theta $ (larger for smaller scattering angle), and some properties of the participating ions or neutral particles (Fernández-Varea et al. 1993). Inelastic scattering reduces the electron energy $W$ by an amount that is used to excite or ionise the collision partners. The corresponding cross-section of this interaction depends on the electron energy loss $\Delta W$ (see Selesnick 2016, and references therein).

**Fig. 14:**
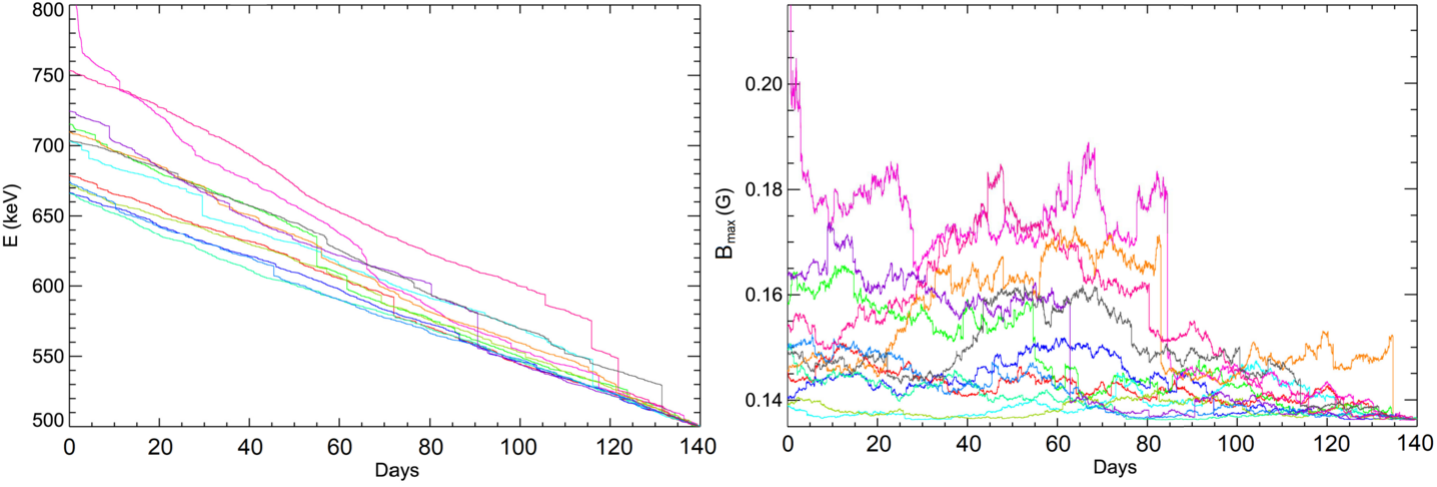
Results of Monte-Carlo simulations of electron trajectories undergoing elastic and inelastic collisions. Left: electron energy; right: magnetic-field strength at the particle mirror points. Electrons are trapped within the inner radiation belts and bounce between the equator with $B(0)\approx 1.39$ G and $B_{\max}$. Adopted from Selesnick (2016) and with permission by John Wiley and Sons

The combination of elastic and inelastic collisions results in decreases in the energies and random variations in the pitch-angles of the affected electrons. The change in pitch-angle can be expressed in terms of the change of the electron mirror-point position, which is quantified in terms of their associated $B_{\max}=B(0)/\sin ^{2}\theta $. If collisional scattering results in increases in $B_{\max}$ to values associated with very low altitudes, the corresponding electrons reach the dense ionosphere at typical altitudes of $\sim 100$ km, where they are finally lost due to the de-trapping through multiple collisions. Figure 14 shows the results of Monte-Carlo simulations of energetic electrons trapped in the inner radiation belt of the Earth’s magnetopshere. The particle energies slowly decrease due to inelastic collisional scattering. When $B_{\max}$ sufficiently increases, particles are lost to the ionosphere. Collisional de-trapping also operates in the solar corona (e.g., Emslie 1978; Kontar et al. 2015) and in the radiation belts of other planets (e.g., Clark et al. 2014; Nénon et al. 2018).

## Electron Diffusion and Energisation Across the Magnetic Field

In this section, we focus our discussion on energy diffusion and spatial diffusion of electrons across tangled magnetic fields.

### Phenomenology of Electron Scattering

There are multiple ways for electrons to interact with fluctuations in the electromagnetic field. Effective *non-resonant scattering* of a given electron on fluctuations in the magnetic field occurs when $k_{\perp}\rho _{\mathrm {e}}\sim 1$, where $k_{\perp}$ is the perpendicular component of the wavevector of the involved field fluctuations (Chen et al. 2001; White et al. 2002). If $k_{\perp}\rho _{\mathrm {e}}\ll 1$, the electron follows adiabatic behaviour in these structures. If $k_{\perp}\rho _{\mathrm {e}}\gg 1$, the small-scale modes average out over one gyro-period and thus do not significantly affect the electron trajectory. Figure 5 depicts these three regimes in the context of turbulent magnetic and electric fields.

In the case of non-resonant scattering, classical Gaussian diffusion models predict that the mean squared displacement of the affected particles is proportional to the elapsed time, i.e.,

21 $$ \langle \Delta x^{2}\rangle \propto \tau , $$

like in the case of Brownian motion, where $\Delta x=x(t+\tau )-x(t)$. A complication arises from the fact that the turbulent fluctuations on which the particles scatter are intermittent and anisotropic (see Sect. 2.1.1). Intermittency and anisotropy of the turbulence lead to anomalous diffusion with

22 $$ \langle \Delta x^{2}\rangle \propto \tau ^{\alpha}, $$

where $\alpha \ne 1$ (Zimbardo et al. 2006; Perri and Zimbardo 2008). Intermittency also causes large jumps in energy space (Levy flights) and thus anomalous energy diffusion (Isliker et al. 2017). Sections 5.2 and 5.3 provide further details on energy diffusion and spatial diffusion.

The transport of electrons is further complicated by resonant and stochastic behaviour. We have introduced resonant wave–particle interactions in the context of particle de-trapping in Sect. 4.4.1. If the fluctuations that participate in these wave–particle interactions propagate (i.e., $\omega _{\mathrm {r}}\ne 0$), they can match the resonance condition with electrons in Eq. (13) leading to efficient energy transfer (Kennel and Engelmann 1966; Rowlands et al. 1966; Lichtenberg and Lieberman 1983). If the fluctuations have sufficient amplitudes, they perturb the gyro-motion significantly and scatter the particles stochastically (Brunetti and Lazarian 2007). Ion-acoustic waves often satisfy $k \lambda _{\mathrm{De}}\sim 1$, which corresponds to wavelengths that are $\sim 20-100$ times less than $\rho _{\mathrm {e}}$ (e.g., Fuselier and Gurnett 1984; Breneman et al. 2013; Wilson III et al. 2021; Vasko et al. 2022). Since these ion-acoustic waves have high frequencies, their phase speed is often comparable to the electron thermal speed, so that a large fraction of thermal electrons can go into Landau resonance with these waves despite their small wavelengths (e.g., Dum et al. 1974; Petkaki et al. 2006). Likewise, Langmuir waves and lower-hybrid waves often occur with wavelengths much less than $\rho _{\mathrm {e}}$ and still affect electrons significantly via Landau resonance (e.g., Cairns and McMillan 2005; Wilson III et al. 2012; Yoon et al. 2016).

### Energy Diffusion

Plasma turbulence creates tangled electromagnetic fields (see Sect. 2.1) and is one of the key mechanisms responsible for efficient particle energisation (Fermi 1949, 1954). Significant numerical efforts are being devoted to the investigation of the interplay between these different mechanisms (Zank et al. 2015a, 2021; Nakanotani et al. 2021; Trotta et al. 2023) in the energisation of particles.

Turbulent fluctuations perform second-order stochastic particle acceleration in the form of a *diffusion* process in velocity space. In this process, some particles gain energy while others lose energy during their interaction with the fluctuating electric field. Since a larger fraction of particles increase their energy rather than decrease their energy, the process induces a net average particle energisation. Stochastic acceleration of relativistic electrons occurs in the intracluster medium and in galaxy clusters (Brunetti et al. 2001; Petrosian 2001; Brunetti and Lazarian 2016; Brunetti and Vazza 2020). Observations of diffuse radio emission, an indirect proxy for energised electrons, suggest that electron energisation takes place both in the turbulent high-$\beta $ plasma of the intracluster medium and in galaxy clusters, where shock waves can induce particle energisation (Stevens et al. 1999; van Weeren et al. 2017). In the intracluster medium, the resultant heating helps to overcome the radiative cooling that affects electrons more than protons as they suffer from radiative losses (Field 1965). Recently, the observation of *radio bridges* connecting two galaxy clusters is interpreted in terms of stochastic acceleration due to turbulence (Brunetti and Vazza 2020), although other mechanisms, such as weak shocks, may contribute as well to particle energisation in the intracluster medium (van Weeren et al. 2017; Ryu et al. 2019).

In-situ observations of electron beams near the terrestrial bow shock and interplanetary shocks suggest that stochastic electron acceleration at plasma shock waves is strongly affected by ripples in the shock surfaces (Lindberg et al. 2023; Jebaraj et al. 2023). Ripples in the shock front enable multiple encounters of the electron beam with the strong gradients of the shock surface (Xu et al. 2025), a process to which also the herringbone structures in radio bursts are attributed (Holman and Pesses 1983; Zlobec et al. 1993). A complementary scenario invokes the interaction of a planar shock front with tangled and meandering magnetic-field lines that are convected over the shock surface (Decker 1993; Jokipii and Giacalone 2007; Guo and Giacalone 2010, 2015). The electrons follow these tangled magnetic-field lines throughout the heliosphere (Moradi and Li 2019; Bian and Li 2021; Li and Bian 2023) and thus also on the upstream (and downstream) sides of shock surfaces. Like in the shock-ripple scenario, this geometry allows for multiple encounters of streaming electrons with the shock and hence iterative acceleration. Given the ubiquity of turbulence and tangled magnetic fields in collisionless plasmas as well as the known sub-structure of shock waves, the interaction of electrons with shock surfaces is likely to be defined by a combination of both scenarios. Further aspects of the energisation of electrons at collisionless shocks are discussed in the article by Amano et al. (2026).

Plasma turbulence generates inhomogeneous and intermittent patches of *coherent structures*, including eddies, flux ropes, and plasmoids separated by intense current sheets and shear layers, across the entire turbulent spectrum (Brunetti and Lazarian 2007; Bruno and Carbone 2013; Matthaeus et al. 2015). These structures mediate particle transport and energisation intermittently, for example, through magnetic reconnection. Under certain conditions, particles are trapped within these coherent structures. These trapped particles are inhibited in their diffusion and experience intense perpendicular energisation in association with the merging or contraction of islands and plasmoids (Ambrosiano et al. 1988; Dmitruk et al. 2003; Drake et al. 2006; Kowal et al. 2011; le Roux et al. 2015; Lemoine 2021, 2022, 2025; Servidio et al. 2016; Lemoine 2019; Trotta et al. 2020; Pezzi et al. 2022).

**Fig. 15:**
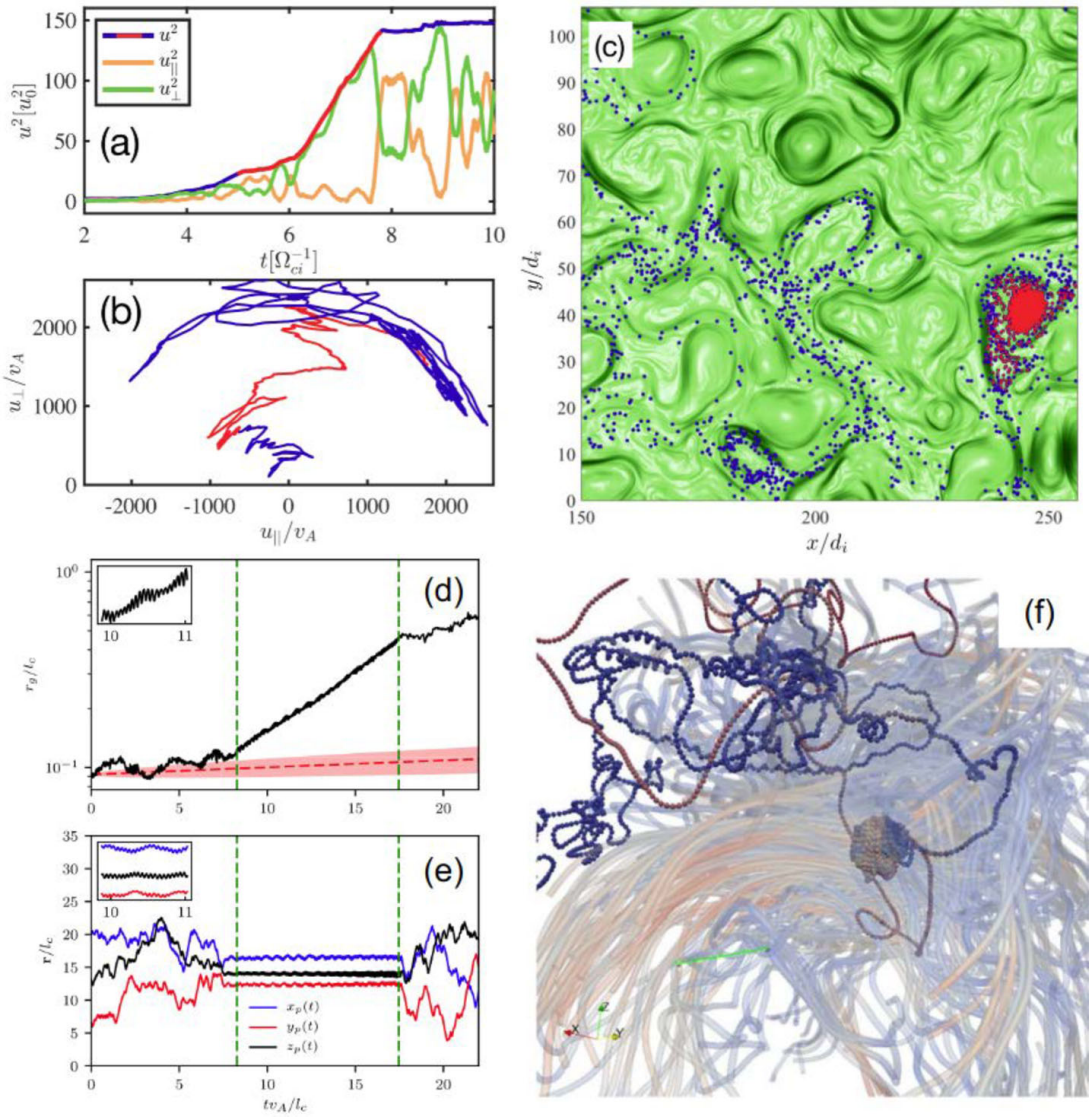
Role of particle trapping for particle energisation in coherent structures. Left: thermal electrons embedded in plasma turbulence modelled with hybrid particle-in-cell simulations (Trotta et al. 2020). Right: energetic protons in incompressible MHD turbulence (Pezzi et al. 2022). In both cases, strong and preferentially-perpendicular energisation is associated with particle trapping in large-scale coherent structures. Trapping phases (red curves in panels (a) and (b), red points in panel (c), and the area between the two vertical green lines in panels (d) and (e)) are preceded and followed by erratic particle motion in the entire computational domain across which particles undergo second-order diffusion in energy as well as isotropisation

Figure 15 illustrates the role of particle trapping in coherent structures as an efficient particle energisation mechanism. The panels on the left-hand side show the results of a two-dimensional hybrid particle-in-cell simulation that investigates the energisation of trans-relativistic test-electrons with gyro-radii of order the ion inertial length (Trotta et al. 2020). The panels on the right-hand side show the results of an MHD simulation focused on fully-relativistic test-protons with gyro-radii in the inertial range of the incompressible MHD turbulence (Pezzi et al. 2022). The electron-energy evolution in panel (a) and the typical velocity-space trajectory in panel (b) indicate that the trapped electrons experience an intense perpendicular energisation during the trapping phase, which is marked with a red curve in panel (a) and red points in panel (c). After they escape from the trapping structures, the electrons cease their energisation and diffuse on constant-energy shells as seen in panel (b). Energetic protons follow a similar evolution. During their erratic motion in the turbulent electromagnetic field, a few protons experience a short period – between the two green vertical lines in panels (d) and (e) – in which they are trapped within a flux-tube-like structure shown in panel (f). During the trapping phase, their energisation is exponential as seen in panel (d) and mostly perpendicular to the magnetic field.

These simulations suggest that particle energisation in a turbulent environment is a multi-stage process in which particle trapping favours a strong energisation across the background magnetic field. This process is mediated by particle trapping and acts in conjunction with other energisation mechanisms, including collisionless shocks and magnetic reconnection (Giacalone and Neugebauer 2008; Karimabadi et al. 2014; Zank et al. 2015b; Nakanotani et al. 2021; Trotta et al. 2020, 2021, 2022, 2023), leading to efficient acceleration of particles in space, astrophysical, and laboratory plasmas.

In the solar corona, which serves as a testbed for particle acceleration, turbulent scattering leads to diffusive transport of flare-accelerated electrons, trapping them in the corona (Emslie et al. 2018). For example, combined X-ray ($<100$ keV) and microwave ($>100$ keV) observations suggest the presence of scattering, leading to an energy-dependent relationship for the turbulence scattering mean free path such that the mean free path is inversely proportional to energy (Musset et al. 2018). Moreover, multiple studies of electron properties, determined from X-ray spectroscopy and imaging (Kontar et al. 2011; Benz 2017), suggest some form of coronal electron trapping. Observational evidence of this trapping includes spectral-index differences (Chen and Petrosian 2012) and ratios of electron-acceleration rates (Simões and Kontar 2013). If flare electrons stream from assumed acceleration sites in the tenuous corona to a dense thick-target chromosphere (Brown 1971), the spectra in the corona and chromosphere are expected to show a spectral-index difference of $\sim 2$ based on the comparison of predictions for thin-target and thick-target bremsstrahlung in these regimes. However, observed differences are often less than 2, possibly indicating electron trapping (Chen and Petrosian 2012), while the electron trapping is more efficient for the highest-energy electrons (Musset et al. 2018). Electron-acceleration rates in the corona compared to the chromosphere indicate that some portion must be trapped in the corona (Simões and Kontar 2013).

In the last few years, after the launch of Solar Orbiter (Müller et al. 2020), stereoscopic flare observations, which view the flare from two different angles, are now possible. This new capability brings multiple opportunities for studies related to flare turbulence and particle acceleration. The Spectrometer/Telescope for Imaging X-rays (STIX; Krucker et al. 2020) on Solar Orbiter, alongside Earth-orbiting X-ray observatories such as the Fermi Gamma-ray Burst Monitor (GBM; Meegan et al. 2009) and the Advanced Space-based Solar Telescope Hard X-ray Imager (ASO-S/HXI; Zhang et al. 2019), observe flare X-ray emission from different locations. In particular, this allows us to probe the angular distributions of flare-accelerated electrons often known as the *electron directivity*. This parameter is a key link to the underlying acceleration and transport processes, and hence, to the role of turbulence and particle diffusion (Jeffrey et al. 2024).

### Spatial Diffusion

As discussed in the previous sections, thermal electrons in non-relativistic space and astrophysical plasmas follow the magnetic-field lines due to their small gyro-radius most of the time. Thus, in the classical case described by Braginskii (1965), perpendicular diffusion is weak (see also Coburn et al. 2026). At small spatial scales $\sim \rho _{\mathrm{e}}$, the energy of the turbulent fluctuations, either pre-existing or self-generated through instability, is typically much less than the turbulent energy at large injection scales $\sim \ell _{\mathrm {c}}$ (see Sect. 2.1.1). However, there is growing evidence that also magnetic fluctuations at small scales suppress parallel thermal diffusion as shown by theoretical and numerical studies (Levinson and Eichler 1992; Pistinner and Eichler 1998; Roberg-Clark et al. 2018; Beckmann et al. 2022; Yerger et al. 2025) and in measurements in the solar wind (Gary et al. 1999; Halekas et al. 2021; Coburn et al. 2024); see also Fig. 1 of Coburn et al. (2026) and the detailed discussion therein.

The spatial transport of energetic electrons is very different from that of thermal electrons due to their large gyro-radii. If their energy is sufficiently large (i.e., $v_{\perp}\sim v\sim c$), their cyclotron-resonant wavenumber according to Eq. (13),

23 $$ k_{\mathrm{e,res}}\sim (\omega _{\mathrm {r}}-n\Omega _{\mathrm {e}})/c \sim n/\rho _{\mathrm {e}}, $$

eventually falls within the inertial range of the plasma turbulence. Galactic cosmic-ray electrons that escape supernova remnants and propagate through the interstellar medium are such a population of energetic particles. As seen in Fig. 4 of Adriani et al. (2023), cosmic-ray electrons have energies up to (at least) $\sim 1$ TeV, although the highest energy of cosmic-ray electrons is still debated (Sudoh and Beacom 2023). Moreover, electron energies are significantly altered by radiative losses in contrast to protons (Cristofari et al. 2021; Evoli et al. 2021; Dörner et al. 2023). Considering the fiducial values of $1\,\mu $G for the magnetic field and 1 cm^−3^ for the particle density in the interstellar medium, the gyro-radius $\rho _{\mathrm {e}}$ of a TeV electron is about $\sim 10^{-3}$ pc and thus much smaller than the correlation length $\ell _{\mathrm {c}}\sim 10$ pc of the turbulence but greater than the proton inertial length $d_{\mathrm {p}}\sim 10^{-6}$ pc. Therefore, the resonant wavenumber of these energetic electrons likely falls in the inertial range of the turbulence in the interstellar medium. At these energies, the energetic electrons diffuse similarly to protons, and the considerations about proton diffusion across the magnetic field also apply to electrons.

In addition to the resonant diffusion mechanisms that depend on the available energy at the scale $k_{\mathrm{e, res}} \sim 1/\rho _{\mathrm {e}}$ (Shalchi 2009), electrons may diffuse due to alternative mechanisms. An efficient particle diffusion mechanism is the large-scale *field-line random walk* (FLRW) of magnetic-field lines that induces particle diffusion across the background magnetic field (Laitinen et al. 2016). This diffusion occurs either homogeneously or ordered on large scales $\sim \ell _{\mathrm {c}}$. Turbulence is capable of suppressing thermal conduction by FLRW to values that are about two orders of magnitude below the Spitzer value in galaxy-cluster flows (Chandran and Cowley 1998). This behaviour is also observed in the case of super-Alfvénic turbulence at the outer scales, i.e., in the case of velocity perturbations at the injection scales that are greater than the Alfvén speed (Lazarian 2006). However, turbulence can also enhance heat conduction, depending on the plasma magnetisation and the turbulence driving (Lazarian 2006), in which case turbulence induces electron advection that provides effective heat diffusivity up to values exceeding the Spitzer value.

Understanding the properties of spatial diffusion perpendicular to the magnetic field is a major challenge that still motivates significant theoretical (Matthaeus et al. 2003; Shalchi et al. 2004; Shalchi 2009, 2010, 2015, 2019, 2021) and numerical research efforts (Casse et al. 2001; Shalchi et al. 2004; DeMarco et al. 2007; Cohet and Marcowith 2016; Pucci et al. 2016; Arendt and Shalchi 2020; Dundovic et al. 2020; Mertsch 2020; Reichherzer et al. 2022; Maiti et al. 2022; Kuhlen et al. 2025). From the theoretical perspective, different theories and models, including classical quasilinear theory and second-order quasilinear theory, are proposed for the calculation of the diffusion coefficient for the resonant diffusion parallel to the magnetic field. The assumption of a particular type of turbulence (composite slab and 2D, three-dimensional isotropic, critically balanced, etc.) allows us to evaluate the pitch-angle diffusion coefficient $D_{\xi \xi}$ (Shalchi 2009; Schlickeiser 2002), which in turn depends on the resonances with the magnetic-field fluctuations at wavenumber $k_{\mathrm{e,res}}\sim 1/\rho _{\mathrm{e}}$. The parallel diffusion coefficient is then directly given by (Schlickeiser 2002)

24 $$ D_{\parallel }= \frac{3 v}{8} \int \limits _{-1}^{+1} \frac{(1-\xi ^{2})^{2}}{D_{\xi \xi}}\,\mathrm {d}\xi , $$

where $\xi $ is the pitch-angle cosine $\xi =\cos \theta = v_{\parallel}/v$.

**Fig. 16:**
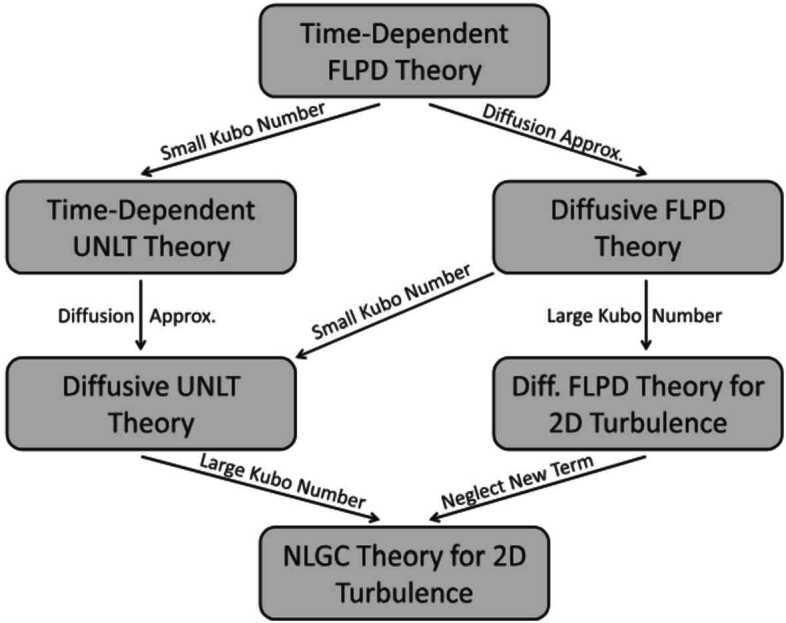
Schematic view of the links between a set of recent theories on perpendicular particle transport. The Kubo number $K = (\ell _{\mathrm {c},\parallel}/\ell _{\mathrm {c},\perp}) (\delta B_{ \perp}/B_{0})$ quantifies the intensity of the turbulent fluctuations modulated with the ratio of the parallel to the perpendicular correlation lengths $\ell _{\mathrm {c},\parallel}$ and $\ell _{\mathrm {c},\perp}$. FLPD: field-line–particle decorrelation, UNLT: unified nonlinear theory, NLGC: nonlinear guiding centre. The diffusive FLPD theory for 2D turbulence includes a new term that contains explicitly the field-line diffusion coefficient (Shalchi 2021). If this term is neglected, the diffusive FLPD theory for 2D turbulence transitions into the NLGC theory for 2D turbulence. From Shalchi (2021)

The parallel diffusion coefficient is one of the key ingredients for the calculation of the perpendicular diffusion coefficient $D_{\perp}$, and different theories and models exist for the evaluation of $D_{\perp}$. These include the *nonlinear guiding-centre* (NLGC; Matthaeus et al. 2003), the *unified nonlinear* (UNL; Shalchi 2010), and the *field-line–particle decorrelation* (FLPD; Shalchi 2021) *theories*. Figure 16 illustrates the links and inter-dependencies between these theoretical frameworks. Most of them rely on a similar methodology which we briefly sketch out below. The goal of all these theories is the calculation of the perpendicular diffusion coefficient $D_{\perp}$ as defined in the Taylor–Green–Kubo (TGK) formulation (Kubo 1957):

25 $$ D_{\perp} = \int \limits _{0}^{\infty }\langle v_{x}(t) v_{x}(0) \rangle \,\mathrm {d}t, $$

where $\langle \dots \rangle $ denotes the ensemble average, and we assume that the background field $\boldsymbol{B}_{0}$ is along the $z$-direction. The derivation procedure is then as follows:

1. The equations of motion are solved in a given magnetic-field configuration to calculate the second-order perpendicular correlation $\langle v_{x}(t) v_{x}(0)\rangle $, usually with the additional assumption that the magnetic-field perturbations are slow and that their component parallel to $\boldsymbol{B}_{0}$ is negligible ($\delta B_{z}=0$). The second-order perpendicular correlation $\langle v_{x}(t) v_{x}(0)\rangle $ can be written as a fourth-order correlation including the parallel speed of the gyro-centre ${\tilde{v}}_{z}$ and the perpendicular magnetic-field perturbations $\delta B_{\perp}$.
2. The fourth-order correlation is separated into two second-order auto-correlations: one for the parallel velocity and one for the perpendicular magnetic-field perturbations.
3. A description for the auto-correlation function of the parallel velocity is then introduced, e.g., an exponential decay. This description relates the auto-correlation function of the parallel velocity with the parallel diffusion coefficient $D_{\parallel}$.
4. Corrsin’s hypothesis for homogeneous turbulence is usually invoked to relate the auto-correlation function of the perpendicular magnetic field to the energy spectrum of the turbulence (Shlien and Corrsin 1974; Tautz and Shalchi 2010).
5. Finally, ensemble averages are calculated by assuming some distribution of particles and a closure for the particle mean displacements (e.g., a diffusive closure).

These steps generally lead to a nonlinear integral equation for $D_{\perp}$ that depends on $D_{\parallel}$ and on the type, intensity, and spectral properties of the magnetic-field turbulence. The above-listed theories have been tested against numerical simulations of charged test-particles in turbulent fields, generated either synthetically (Dundovic et al. 2020) or through MHD simulations (Cohet and Marcowith 2016; Maiti et al. 2022). A still-debated aspect concerns whether the perpendicular diffusion coefficient has the same energy dependence as the parallel diffusion coefficient for $\rho _{\mathrm{e}}<\ell _{\mathrm {c}}$. Indeed, different models predict that the parallel and perpendicular diffusion coefficients should scale similarly with particle energy $W$; however, there is growing evidence for different energy dependencies of $D_{\parallel}$ and $D_{\perp}$ in numerical simulations (DeMarco et al. 2007; Dundovic et al. 2020; Kuhlen et al. 2025).

An additional fundamental problem is related to the nature of the mechanisms responsible for parallel diffusion. Resonant diffusion critically depends on the power in the fluctuations at the resonant scale $k_{\mathrm{e,res}}$. Diffusion is weakened when the power is weak or absent at this scale, as it is the case for electrons with low to intermediate energies in anisotropic Alfvénic or slow-magnetosonic turbulence. Fast-magnetosonic turbulence can partially overcome this issue since it cascades isotropically (Cho and Lazarian 2002). However, the spectrum of fast-magnetosonic turbulence is affected by various damping mechanisms depending on the properties of the background medium, such as the plasma-$\beta $.

Other processes that contribute to particle diffusion when resonant diffusion is weak include FLRW (Pezzi and Blasi 2024) and small-scale magnetic-field curvature (Kempski et al. 2023; Lemoine 2023; Kempski et al. 2025). The FLRW-driven diffusion provides an energy-independent diffusion coefficient which adds to the classical resonant diffusion for galactic cosmic rays (Pezzi and Blasi 2024). Curvature-driven diffusion instead provides an energy-dependent diffusion coefficient with a scaling that is similar to that predicted by standard resonant diffusion. Further efforts are still needed to explore the importance of these mechanisms depending on the key parameters of the turbulence (e.g., $\delta B/B_{0}$ or the level of intermittency) and to understand whether they induce features in the cosmic-ray energy spectrum.

The microphysics of energetic-particle transport has vast applications in astrophysics. The perpendicular and parallel diffusion coefficients serve as inputs for the solution of advection-diffusion transport equations, such as the Parker equation and the focused-transport equation (for a review, see Zank 2014). These equations describe particle transport in various contexts, including the galactic-cosmic-ray transport in the interstellar medium (Skilling 1971; Schlickeiser 2002; Blasi 2013; Dörner et al. 2023) and the energetic-particle transport in the heliosphere (Parker 1965; Gordovskyy et al. 2014; le Roux et al. 2015; Zimbardo et al. 2017; Pezzi et al. 2021; Engelbrecht et al. 2022; Wijsen et al. 2022).

## Conclusions

The scales associated with kinetic electron physics are typically the smallest characteristic scales associated with collective behaviour in plasmas. The electron gyro-radius $\rho _{\mathrm {e}}$ is often much smaller than the scales associated with variations in the magnetic field, even when the field is highly tangled. In these cases, the electrons follow the magnetic-field lines, which allows us to understand and use them as tracers of magnetic connectivity in plasma systems. Since electron heat transfer is strongest along the magnetic field, also heat transfer does not follow straight lines in these tangled field configurations.

Tangled magnetic fields can lead to drifts, trapping, and the scattering of electrons. Trapping occurs in localised regions of low field magnitude when particles experience consecutive reflections due to the mirror force. There are two main pathways for electrons to experience scattering: (a) via resonant wave–particle interactions when electrons experience acceleration through quasi-steady wave electric fields in their own reference frame; or (b) when the gyro-radii of the electrons are comparable to the scale of variations in the magnetic field, leading to spatial diffusion along and across the field.

Trapping, wave–particle interactions, and cross-field diffusion are often related and mutually depend on each other. Cosmic magnetic fields are almost always inhomogeneous and tangled, from the phase of magnetogenesis to the turbulent evolution of evolved plasmas. Therefore, electron transport is generally the result of a complex interplay of streaming along tangled field lines, gyro-centre drifts, collisions, trapping in inhomogeneous structures, and scattering on electric and magnetic fluctuations. All of these processes impact the large-scale behaviour of the plasma, for instance, through direct modifications of the electron fluid moments, inter-species coupling, and anomalous resistivity.

In-situ observations in space plasmas open a unique window into the understanding of this complex interplay between large-scale and small-scale processes through the simultaneous multi-scale measurement of electromagnetic fields and electron velocity distribution functions. With the help of stereoscopic and polarisation observations from Solar Orbiter and PADRE, we are now able to infer the electron pitch-angle distribution in solar flares. These observations provide a new diagnostic for the study of electron transport, the presence and nature of turbulence, and the structure of tangled magnetic fields in the flaring corona and similar cosmic plasma environments. Extrapolation from these heliospheric plasmas provides us with the opportunity to understand the transport of electrons in tangled fields across the Universe. As a first step and in dialogue with astrophysical observations, we must understand how tangled fields are in different cosmic plasmas beyond the heliosphere. By extrapolation from our knowledge about heliospheric plasmas, we must then quantify the relative importance of trapping, wave–particle interactions, and diffusion in these environments.

The inclusion of complex electron transport in global plasma models is a central challenge for plasma theory. For instance, simulations of magnetogenesis, cosmic dynamos, and the tangling of the magnetic field must account for electron-kinetic effects self-consistently. As another example, electron heat conduction plays an important role in the magnetothermal instability. If heat flux is suppressed, for example, by resonant wave–particle interactions, this instability is quenched, with profound implications for cosmic dynamos. Simulations also show that particle energisation in turbulent fields is a multi-stage process that involves electron-kinetic physics on all relevant scales. Trapping in islands and other localised field structures has emerged as an efficient energisation process. However, it is still not understood how this process compares to other energisation mechanisms. The answer to this fundamental problem involves not just the characterisation of the nature and occurrence of trapping structures. It also requires a careful analysis of the interactions responsible for the trapping and de-trapping of electrons as well as the involved feedback loops between kinetic-scale and global processes.

Electron transport in tangled magnetic fields is an important contemporary science challenge for the fields of heliophysics and astrophysics. Due to the complexities associated with the self-consistent interdependencies between kinetic-scale and global processes in this context, a truly multi-disciplinary approach of combining in-situ plasma measurements, remote-sensing plasma observations, and plasma theory and simulations is required. This approach must bridge from detailed in-situ measurements of the smallest characteristic plasma scales to the understanding of the largest plasma systems in the Universe.

## Footnotes

1 Equation (1) also applies in the relativistic case as long as $m_{\mathrm {e}}$ accounts for the relativisic correction $m_{\mathrm {e}}=\gamma m_{0\mathrm {e}}$, where $\gamma =1/\sqrt{1-v^{2}/c^{2}}$ is the Lorentz factor and $m_{0\mathrm {e}}$ is the rest mass of an electron. In our convention, $\Omega _{\mathrm {e}}<0$.

2 In general, $\Omega _{\mathrm {e}}\propto 1/\gamma $, where $\gamma =1/\sqrt{1-v^{2}/c^{2}}$, so that electrons at high (relativistic) energies have a sufficiently small $|\Omega _{\mathrm {e}}|$ to resonate with low-frequency waves such as electromagnetic ion-cyclotron waves (Kersten et al. 2014; Ni et al. 2015).
